# Regional growth and atlasing of the developing human brain

**DOI:** 10.1016/j.neuroimage.2015.10.047

**Published:** 2016-01-15

**Authors:** Antonios Makropoulos, Paul Aljabar, Robert Wright, Britta Hüning, Nazakat Merchant, Tomoki Arichi, Nora Tusor, Joseph V. Hajnal, A. David Edwards, Serena J. Counsell, Daniel Rueckert

**Affiliations:** a Biomedical Image Analysis Group, Department of Computing, Imperial College London, London SW7 2AZ, United Kingdom; b Centre for the Developing Brain, Division of Imaging Sciences and Biomedical Engineering, King's College London, London SE1 7EH, United Kingdom; c Clinic of Pediatrics I, Department of Neonatology, University Hospital Essen, D-45122 Essen, Germany

## Abstract

Detailed morphometric analysis of the neonatal brain is required to characterise brain development and define neuroimaging biomarkers related to impaired brain growth. Accurate automatic segmentation of neonatal brain MRI is a prerequisite to analyse large datasets. We have previously presented an accurate and robust automatic segmentation technique for parcellating the neonatal brain into multiple cortical and subcortical regions. In this study, we further extend our segmentation method to detect cortical sulci and provide a detailed delineation of the cortical ribbon. These detailed segmentations are used to build a 4-dimensional spatio-temporal structural atlas of the brain for 82 cortical and subcortical structures throughout this developmental period. We employ the algorithm to segment an extensive database of 420 MR images of the developing brain, from 27 to 45 weeks post-menstrual age at imaging. Regional volumetric and cortical surface measurements are derived and used to investigate brain growth and development during this critical period and to assess the impact of immaturity at birth. Whole brain volume, the absolute volume of all structures studied, cortical curvature and cortical surface area increased with increasing age at scan. Relative volumes of cortical grey matter, cerebellum and cerebrospinal fluid increased with age at scan, while relative volumes of white matter, ventricles, brainstem and basal ganglia and thalami decreased. Preterm infants at term had smaller whole brain volumes, reduced regional white matter and cortical and subcortical grey matter volumes, and reduced cortical surface area compared with term born controls, while ventricular volume was greater in the preterm group. Increasing prematurity at birth was associated with a reduction in total and regional white matter, cortical and subcortical grey matter volume, an increase in ventricular volume, and reduced cortical surface area.

## Highlights

• A novel methodology is proposed for delineating the cortical ribbon.
• Regional brain growth is assessed in the developing preterm brain.
• We investigate the effect of prematurity on brain growth and cortical development.
• A spatio-temporal neonatal atlas is constructed with 82 brain structures.

## Introduction

The incidence of preterm birth continues to rise, with an estimated 14.9 million infants (representing 11.1% of all births) delivered worldwide each year (Blencowe et al., 2012). Insights into impaired neurodevelopment in these vulnerable infants have been gained from magnetic resonance (MR) imaging studies assessing brain development during the period between preterm birth and the normal time of birth. Brain volumetric and cortical surface measurements provide important information regarding brain development and have the potential to predict long-term neurodevelopmental performance (Peterson et al., 2003, Counsell et al., 2008, Thompson et al., 2008, Rathbone et al., 2011, Boardman et al., 2010). Previous studies in preterm infants have demonstrated reduced brain volume (Peterson et al., 2003, Inder et al., 2005, Thompson et al., 2007, Ball et al., 2012) and decreased cortical surface area (Ajayi-Obe et al., 2000, Kapellou et al., 2006), which are related to subsequent adverse neurodevelopmental outcome (Kapellou et al., 2006, Rathbone et al., 2011). However, studies to date have focused on characterising brain tissue volumes or volumes of large subcortical structures. In addition, sample sizes have usually been small, over a limited age range and detailed regional brain growth has not been studied (Hüppi et al., 1998, Murphy et al., 2001, Peterson et al., 2003, Inder et al., 2005, Prastawa et al., 2005, Mewes et al., 2006, Nishida et al., 2006, Zacharia et al., 2006, Gilmore et al., 2007, Song et al., 2007, Thompson et al., 2007, Xue et al., 2007, Anbeek et al., 2008, Dubois et al., 2008a, Dubois et al., 2008b, Pienaar et al., 2008, Rodriguez-Carranza et al., 2008, Yu et al., 2010, Cardoso et al., 2013, Wang et al., 2012, Moeskops et al., 2013, Moeskops et al., 2015).

Due to the lack of manually segmented atlases, previous automatic methods (Peterson et al., 2003, Mewes et al., 2006, Gilmore et al., 2007, Thompson et al., 2007) did not segment deep grey matter structures and parcellated cortical grey matter (CGM) and white matter (WM) regions according to arbitrary linear parcellations which did not reflect regional anatomy. The first regional atlases of the brain were manually delineated by Gousias et al. (2012) that define 50 brain regions in 20 term-equivalent neonatal brains. In Makropoulos et al. (2014) we presented the first study to automatically segment the neonatal brain from the early preterm period to term-equivalent age, into 50 structures with the use of these atlases (82 regions when the WM/CGM regions defined in the atlases are further subdivided into the corresponding WM and CGM parts).

Accurate delineation of the cortex in the neonatal brain is challenging due to partial volume effects and limits in MR imaging resolution. The interior cortical boundary is difficult to delineate as CGM–WM partial volume (PV) can lead to overestimation of the segmented CGM. Furthermore, accurate delineation of the exterior cortical boundary is challenging as the complexity of the cortical surface in conjunction with limits in the MR imaging resolution renders the sulci delineation problematic. Only a few segmentation approaches focus on delineating the cortical ribbon in terms of morphology in the neonatal population (Xue et al., 2007, Wang et al., 2011, Wang et al., 2013). However, surface measurements are affected from undetected sulci, leading to increased cortical thickness estimates.

These technical challenges have resulted in a limited number of studies assessing cortical surface measurements in the neonatal brain. Cortical surface area measurements have been reported in Kapellou et al. (2006), Xue et al. (2007), Dubois et al., 2008a, Dubois et al., 2008b, Pienaar et al. (2008), Rodriguez-Carranza et al. (2008), Rathbone et al. (2011), Lefevre et al. (2015), Moeskops et al. (2015) and curvature measurements/gyrification indices in Xue et al. (2007), Pienaar et al. (2008), Rodriguez-Carranza et al. (2008), Moeskops et al. (2013), Wang et al. (2013), Lefevre et al. (2015), Moeskops et al. (2015).

This study:

• proposes a novel methodology for delineating the cortical ribbon.
• assesses regional brain growth in the developing preterm brain.
• constructs a 4-dimensional spatio-temporal structural atlas with 82 labelled structures of the developing brain.
• investigates the effect of prematurity on regional brain growth and cortical development.

## Methods

Permission for MR imaging was granted by Queen Charlotte's and Hammersmith Hospitals Research Ethics Committee (09/H0707/98, 07/H0704/99 & 07/H0707/101) and written parental consent was obtained prior to imaging.

### Subjects

MPRAGE and T2-weighted MR imaging was performed on a Philips 3 Tesla Achieva system sited on the neonatal intensive care unit using an 8 channel phased array head coil. A cohort of 380 infants recruited from the Neonatal Intensive Care Unit at Queen Charlotte's and Hammersmith Hospitals was used in this study. Exclusion criteria for this study were focal abnormalities on MR imaging as determined by an expert perinatal neuro-radiologist. Out of the 380 infants in the cohort (467 images), 14 infants (19 images) were excluded due to abnormality: 4 infants with cystic periventricular leukomalacia (PVL) (7 images), 5 with Hemorrhagic Parenchymal Infarction (HPI) (6 images), 2 with pseudocysts (3 images), 1 with cerebellar haemorrhage, 1 with multiple white matter infractions and 1 with multiple cystic lesions following meningitis. 28 images were further excluded due to motion artefacts. The resulting dataset was used for the analysis in this work.

We studied 338 infants (298 preterm infants and 40 healthy term born infants) who were born at a median (range) gestational age (GA) of 30 (23^+ 2^–42) weeks. 49 preterm infants had chronic lung disease (CLD), 49 patent ductus arteriosus (PDA) and 32 culture positive sepsis. 66 preterm infants were scanned on 2 occasions and 8 on 3 occasions, resulting in 420 MR imaging data-sets. The interval between scans ranges from 6 days to 16 weeks, with a median of 8^+ 1^ weeks. The term born infants included in this study were scanned only once. The median PMA at imaging was 39^+ 1^ (27^+ 1^–44^+ 6^) weeks and postnatal age at scan 5^+ 3^ (0–19^+ 5^) weeks. There were no significant differences in GA at birth, incidence of sepsis, PDA or CLD between the study group and those excluded due to focal lesions or motion corrupt images. There was no significant difference in age at scan between the study group and those with focal lesions, however the motion corrupted images were acquired at a significantly younger age at scan (*p* = 0.0016). The characteristics of the term-born and preterm infants are presented in Table 1.

### MR imaging acquisition

All examinations were supervised by a paediatrician experienced in MR imaging procedures. Preterm infants at term age were sedated with oral chloral hydrate (25–50 mg/kg) prior to scanning and pulse oximetry, temperature and electrocardiography were monitored. Ear protection was used, comprising earplugs moulded from a silicone-based putty (President Putty, Coltene Whaledent, Mahwah, NJ, USA) placed in the external auditory meatus and neonatal earmuffs (MiniMuffs, Natus Medical Inc., San Carlos, CA, USA). T2-weighted images (TR 8670 ms; TE 160 ms; flip angle 90°; slice thickness 2 mm acquired with an overlap of 1 mm; in plane resolution 0.86 × 0.86 mm) were used for segmentation and cortical analysis.

### Data analysis

#### Image segmentation

The T2 images were segmented with the pipeline presented in Makropoulos et al. (2014). The algorithm is based on an Expectation–Maximization (EM) scheme similar to Van Leemput et al. (1999) with a spatial prior term and an intensity model of the image. Spatial priors of the structures are obtained by averaging the warped labels from the 20 manually segmented atlases of Gousias et al. (2012). Image intensities are modelled with a Gaussian Mixture Model (GMM). Proposed adaptations of the segmentation model limit the influence of intensity in the delineation of structures with very similar intensity profiles. An extensive validation was performed to verify the robustness of the algorithm at different ages of the developing neonatal brain. This method allows regional brain growth in neonates to be assessed in an automatic and reproducible way. We refer the reader to Makropoulos et al. (2014) for more details on the individual parts of the pipeline. Table 2 presents the automatically parcellated regions.

**Fig. 1:**
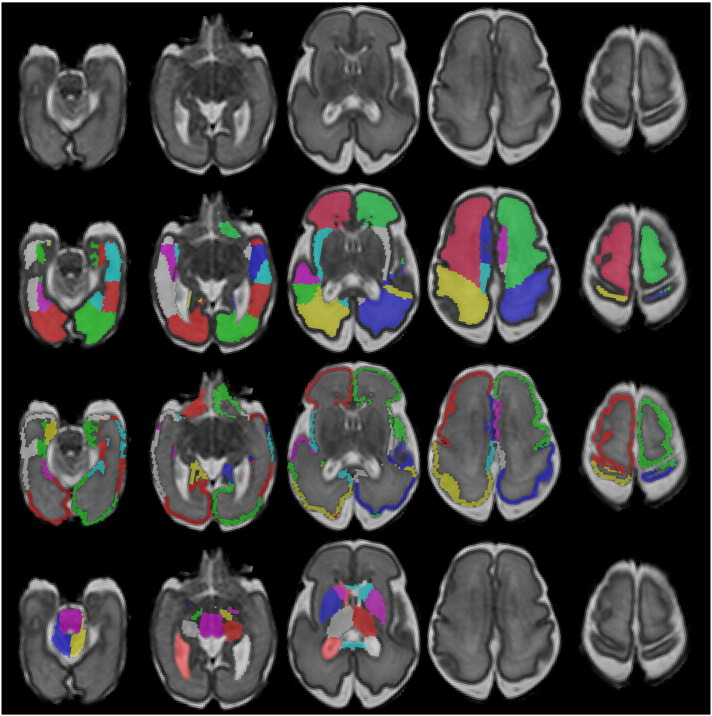
Example segmentation of a neonatal MRI acquired at 28 weeks PMA at scan with the 82 labels overlaid (second row: WM labels, third row: CGM labels, fourth row: subcortical GM labels and ventricles).

**Fig. 2:**
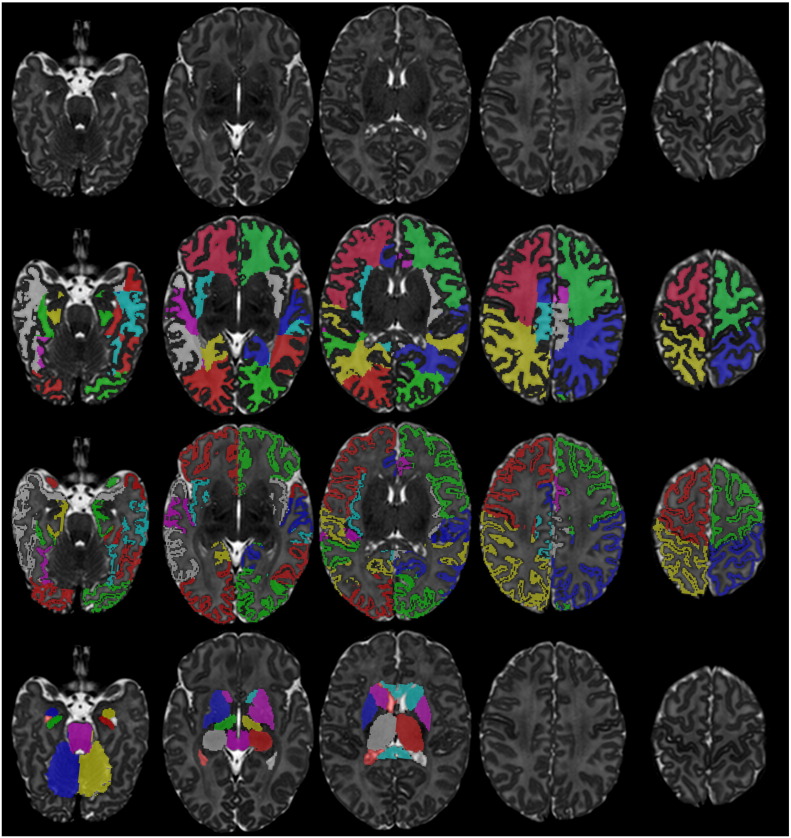
Example segmentation of a neonatal MRI acquired at 44 weeks PMA at scan with the 82 labels overlaid (second row: WM labels, third row: CGM labels, fourth row: subcortical GM labels and ventricles).

In the next sections we present two extensions for the detailed delineation of the cortical ribbon in the neonatal brain. Examples of the proposed segmentation technique applied to an early preterm and term-equivalent brain can be seen in Fig. 1, Fig. 2 respectively.

#### CGM–WM partial volume correction

**Fig. 3:**
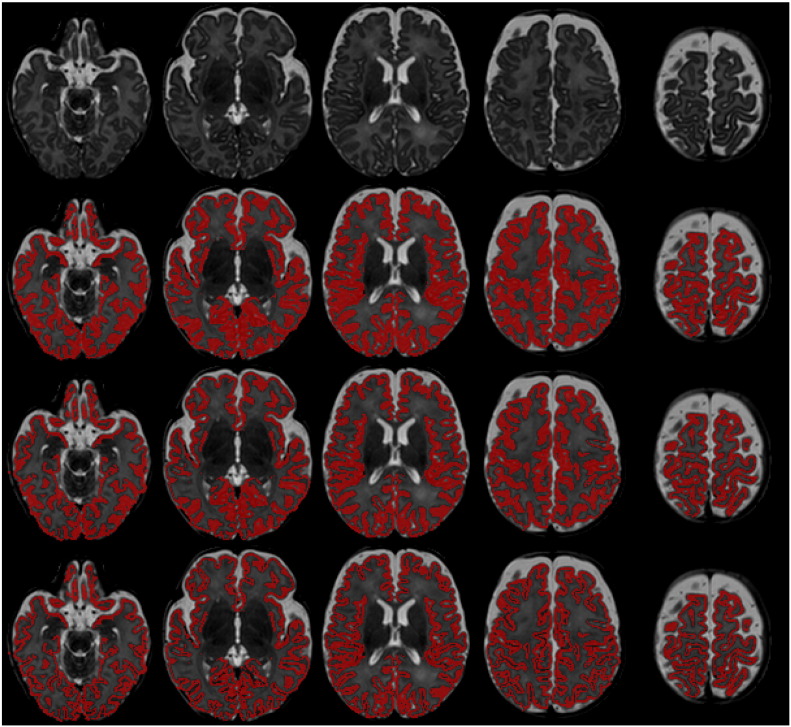
Example segmentations of a neonatal MRI acquired at 44 weeks PMA at scan (A). B presents the original segmentation with the standard Gaussian Mixture Model. C is obtained with the CGM–WM Partial Volume correction, reducing the CGM oversegmentation. D illustrates the final segmentation of the cortex after the sulci delineation.

Due to the partial volume between WM and CGM at the interface between the two tissues, automatic techniques tend to overestimate the CGM volume (Išgum et al., 2015). Fig. 3 depicts this effect. The voxels between WM and CGM have an intermediate intensity and it is difficult to attribute them to either tissue. A Gaussian Mixture Model (GMM) with single classes for WM and CGM tends to overestimate the CGM extent. To account for this effect in Makropoulos et al. (2012) we implemented a partial volume correction for the CGM–WM boundary. In this study, an additional class is added to the GMM as a partial volume class between CGM and WM. Once the EM scheme has converged, the PV class is merged with the WM class to reduce the CGM overestimation and enhance the WM tissue estimate.

#### Sulci detection and enhancement

**Fig. 4:**
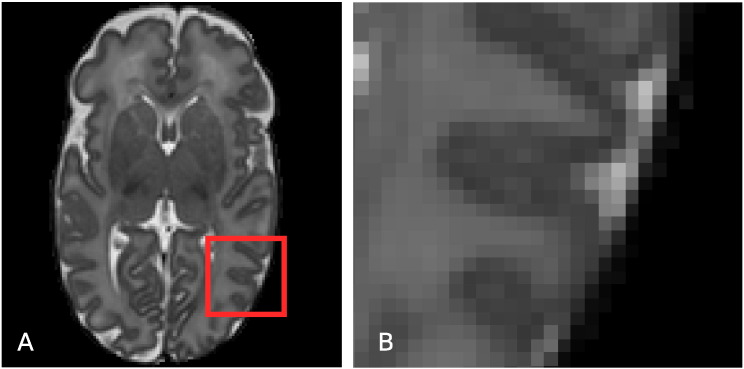
Axial slice of a T2-weighted MRI (A) and magnified region of the cortex (B). Due to PV effects, the CSF inside the cortical sulci is often hard to discriminate, and consequently delineate with intensity-based segmentation techniques. Especially in areas where cortical gyri “touch” each other there is often very little evidence, in terms of intensity, of the CSF inside the sulcus.

Delineation of the sulci in the neonatal cortex is difficult due to the limited resolution that leads to partial volume effects in the exterior cortical surface (see Fig. 4). Here, we present a novel approach for sulci enhancement for the neonatal images, based on the assumption that cortical thickness at sulci locations should be similar over a local neighbourhood of the cortical ribbon.

**Fig. 5:**
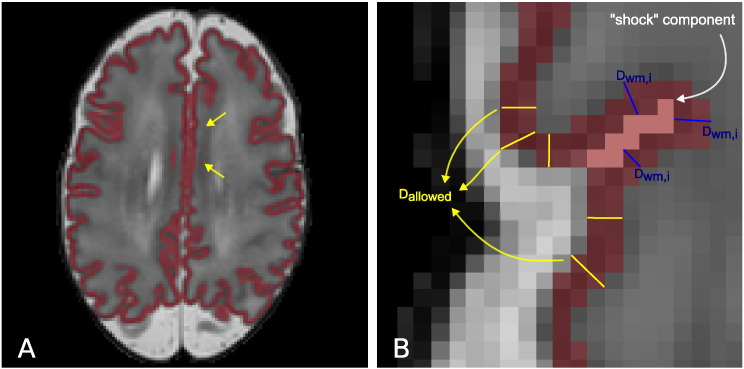
A: T2 with the cortical segmentation overlaid. The arrows show parts of the cortical ribbon connected across the two hemispheres in the midsection of the brain. B: Example shock points (in pink) detected for the cortical segmentation (in red). Shock voxels are labelled as CSF if their distance *D*_*WM*,*i*_ to the WM is larger than *D*_*allowed*_. *D*_*allowed*_ is estimated from neighbouring parts of the cortical ribbon with streamlines that do not cross shock points (yellow lines).

Sulci detection is performed in a way similar to Han et al. (2004). The CGM–WM interface (interior cortical surface) is iteratively propagated with the fast marching method with a speed function derived from the cerebrospinal fluid (CSF) posterior obtained with EM. Shock points are then detected where two sulcal banks merge in the propagated surface as in Han et al. (2004). Due to the limited resolution, the cortical ribbon in the two hemispheres may appear to be connected in different parts of the midsection of the brain (an example can be seen in Fig. 5.A). A second type of shock points is also added here for neighbouring CGM voxels from different hemispheres.

Having detected the shock points, Han et al. (2004) perform morphological thinning to create a thin layer, one voxel thick, of CSF that splits the two sulcal banks apart. However, defining the CSF inside the sulcus to be one voxel wide might lead to incorrect cortical thickness estimates at points in the sulcal banks. As can be seen in Fig. 5.B, since the cortical thickness is estimated between the CGM–WM interface and the CGM–CSF interface, the width of the layer chosen for the shock points has a direct effect on the cortical thickness in the sulcal regions.

**Fig. 6:**
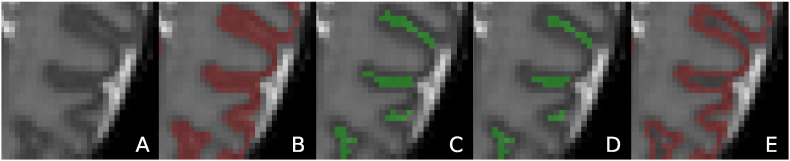
Sulci detection and enhancement. The cortical segmentation of the MRI in A is presented in B and E before and after the sulci delineation. Shock voxels detected are illustrated in C. The voxels that are finally labelled as CSF (sulci enhancement) are shown in D.

In this work the shock voxels are attributed to CSF only if their distance to the CGM–WM interface–the potential thickness if they belong to CGM–is larger than the cortical thickness of neighbouring parts of the cortical ribbon. Voxelwise cortical thickness is estimated as described in Jones et al. (2000). The sulcal points are thus prevented from thickness inconsistent with the rest of the cortex, since their cortical thickness is approximated based on the thickness of neighbouring parts in the cortical ribbon. Further details of the proposed sulci correction method can be found in Appendix A. An illustration of the method is presented in Fig. 6.

#### Cortical surface reconstruction

**Fig. 7:**
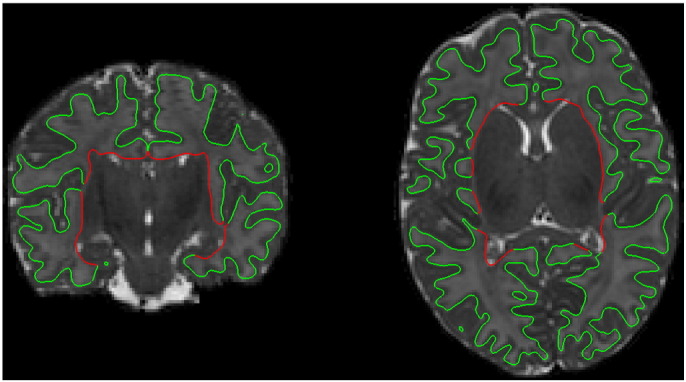
Example cortical surface of a neonate at 44 weeks PMA at scan. The red part of the surface that corresponds to the WM – deep GM boundary is excluded from the cortical surface measurements.

**Fig. 8:**
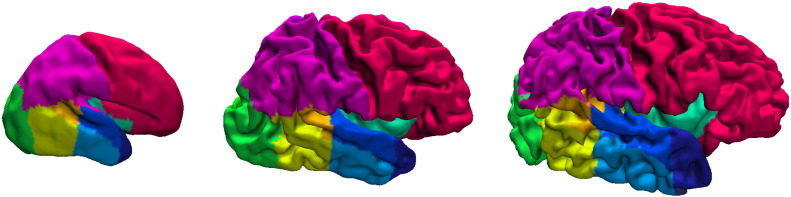
Example cortical surfaces for neonates at 28, 36 and 44 weeks PMA at scan with the labels overlaid.

Cortical surface meshes were obtained by triangulation of the CGM–WM isosurface with the marching cubes algorithm (Lorensen and Cline, 1987). The CGM binary mask was initially blurred with a Gaussian kernel of 1 mm standard deviation to avoid a blocky surface due to the limited resolution. Laplacian smoothing was applied to the surfaces (Herrmann, 1976) to improve the mesh quality. The surface region that belongs to the boundary between WM and deep GM was excluded from the cortical surface (see Fig. 7). Example cortical surfaces are presented in Fig. 8. Topological correction of the surfaces was not addressed. The interior cortical surface does not suffer from the problem of merging gyri and therefore does not present major topological defects.

#### Spatiotemporal structural atlas construction

Structural information of probabilistic brain atlases is constructed by averaging segmentations of different brains in the same coordinate space, in order to account for the anatomical variability in the brain. This section describes the construction of the first regional spatio-temporal structural atlas for the neonatal brain with a methodology similar to Kuklisova-Murgasova et al. (2011), and Serag et al. (2012). As in Kuklisova-Murgasova et al. (2011), and Serag et al. (2012), the segmentations are averaged with a non-parametric kernel regression according to the age at scan of the subjects.

The spatio-temporal template of Serag et al. (2012) is used as the coordinate space of the atlas. This template defines mean brain images for 28 to 44 weeks post-menstrual age at scan (with a week interval). The derived atlas is defined in the same age range. The segmentations of the 420 T2 images are warped to the space of the template according to the age at scan of each subject. In order to enforce consistency of the atlas in the time domain (different ages of the template), each segmentation is warped to the mean images in the range [*a* − 3,*a* + 3], where *a* is the rounded age at scan of the subject in weeks. The transformations are calculated with non-rigid registration of the subject's T2 image to the corresponding mean images of the template. The non-rigid registration is carried out using free-form deformations with control point grid spacings of 20 mm, 10 mm, 5 mm and 2.5 mm and normalized mutual information (NMI) as similarity measure Rueckert et al. (1999). Having computed the transformations for all the subjects, the age-dependent probability map *P*_*k*,*t*_ of each structure *k* at time *t* of the atlas can be computed as:

(1) $$P_{k,t}=\frac{\sum_{s=1}^{S}\left(wt_{s},t\right)M_{s,k}\circ T_{s,t}}{\sum_{s=1}^{S}\left(wt_{s},t\right)}$$

where *s* denotes the different subjects (*S* = 420 in total). *M*_*s*,*k*_ is the binary mask of structure *k* from the segmentation of subject *s*. *M*_*s*,*k*_ is warped (∘) under the transformation *T*_*s*,*t*_ of *s* to the mean image of the template at age *t*. *w*(*ts*,*t*) is an age-dependent weight of subject *s* according to how closely the age *t_s_* of the subject matches the age *t* of the atlas. The weight is defined according to a Gaussian kernel:

(2) $$w\left(t_{s},t\right)=\frac{1}{\sigma_{w}\sqrt{2\pi }}e^{\frac{-{\left(t_{s}-t\right)}^{2}}{2\sigma_{w}^{2}}}$$

where *σ_w_* is set to 1 week. The probabilistic atlas at each age *t* is then defined as the union of the probability maps at the corresponding age: *P_t_* = {*P*_*k*,*t*_}.

A maximum-probability version of the atlas at time *t* is further constructed by assigning the structure with the maximum probability to each voxel at age *t*:

(3) $$P_{\max,t}=\;\arg\max_{k}P_{k,t}$$

**Fig. 9:**
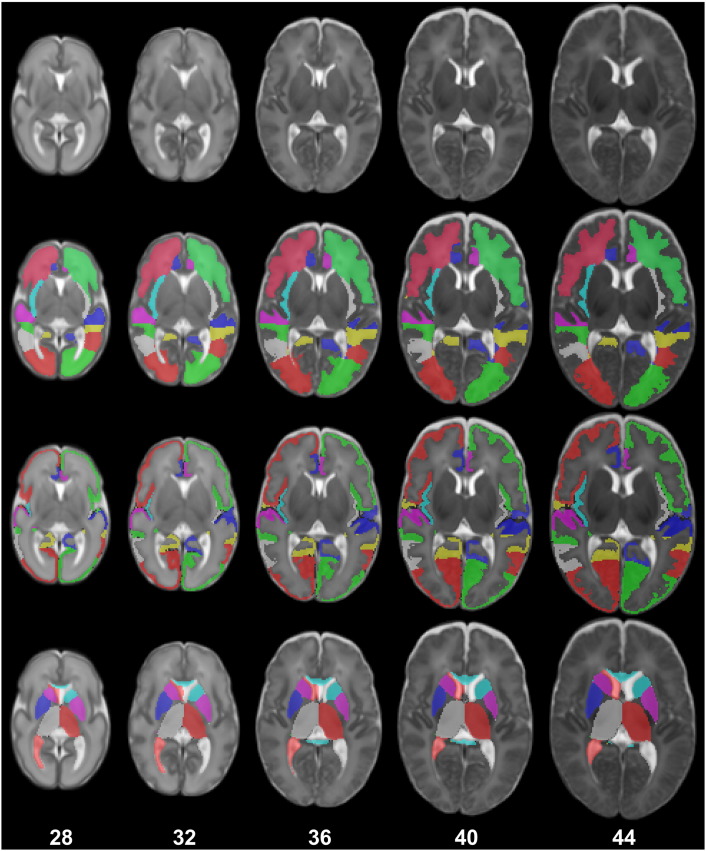
The maximum probability structural atlas shown at different ages. The structures of the atlas (second row: WM structures, third row: CGM structures, fourth row: subcortical GM structures and ventricles) are defined in the coordinate space of the spatio-temporal template of Serag et al. (2012) (first row).

**Fig. 10:**
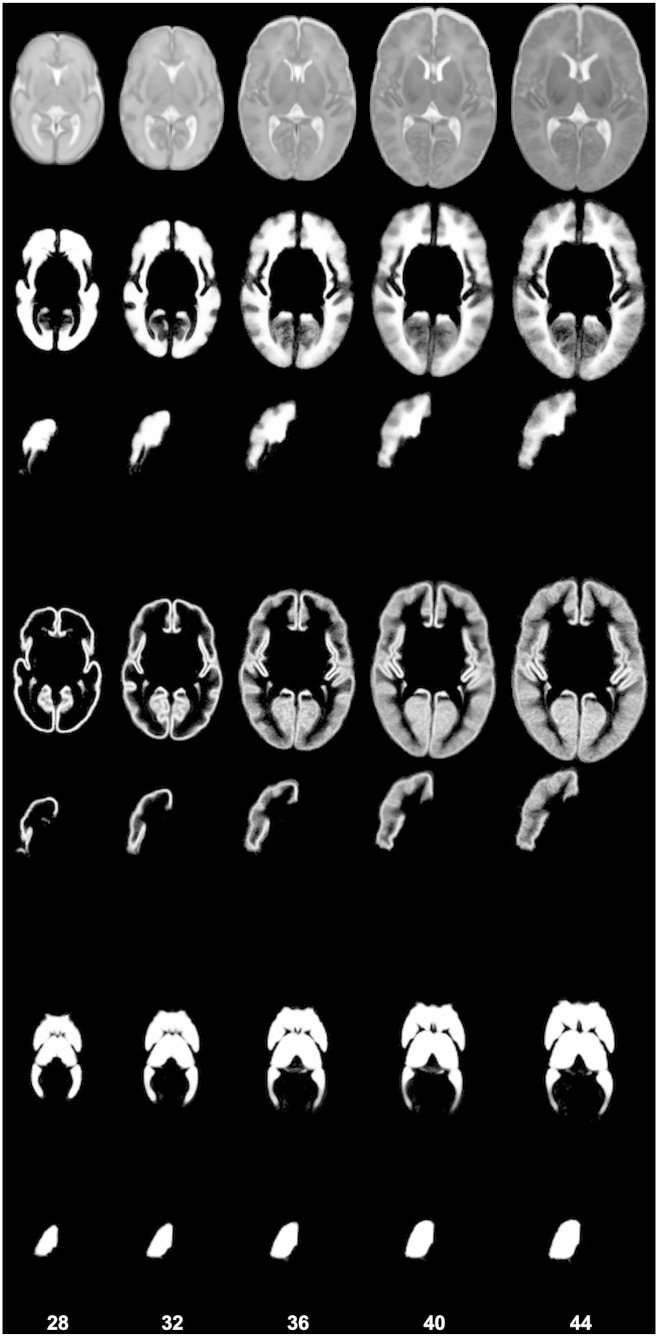
The probabilistic structural atlas shown at different ages. The following probability maps are displayed (second-seventh row): WM (sum of the probability maps of the WM structures), right frontal lobe WM, CGM (sum of the probability maps of the CGM structures), right frontal lobe GM, subcortical GM and ventricles (sum of the probability maps of the subcortical GM structures and the ventricles), right thalamus. The probabilistic structural atlas is defined in the coordinate space of the spatio-temporal template of Serag et al. (2012) (first row).

The constructed structural atlas incorporates the 82 structures of the brain, the CSF, the intra-cranial and the extra-cranial background. Illustrations of the probabilistic and maximum-probability versions of the atlas are presented in Fig. 9, Fig. 10. The atlas is publicly available from http://brain-development.org/.

### Measurements of the brain

#### Volumetric measurements

Absolute and relative volumes of the tissues and 82 structures of the brain were measured directly from the segmentations. Relative volume was determined as the ratio of the structure volume to the total brain volume (excluding the CSF and ventricles).

#### Cortical surface measurements

Surface area and curvature measures of the cortex were computed from the cortical surface meshes. A number of curvature measures were adopted from Rodriguez-Carranza et al. (2008) with T-normalization, effectively normalized according to $T=\frac{3\;\mathrm{volume}}{\mathrm{surface}\;\mathrm{area}}$, that are invariant to the surface area of the brain. This allows comparison of brains with different sizes, as is the case of the developing neonatal brain. The curvature measures included in this study are: global curvedness, mean curvature *L*^2^ norm and Gaussian curvature *L*^2^ norm. Their formulation is presented in Table 3. Regional cortical surface measurements were measured based on the segmented CGM structures. The cortical labels were propagated to the surface meshes. Each vertex of the mesh was labelled with the closest CGM structure in the 3-dimensional space.

### Statistical analysis

We determined the centiles of the volumetric and surface measurements with increasing age at scan for the preterm datasets. Correlations with the age at scan were calculated to investigate premature brain development. The measurements were assessed individually according to the Pearson correlation coefficient and adjusted *R*^2^ values from fitting a linear model to the data. Since the relationship between regional brain volumes and brain development may not always be characterised with a linear model, we additionally use a Gompertz-like function to model this relationship, similar to Wright et al. (2014). We compare the fit of the two models according to the sum of squares error (SSE). The Gompertz function used in this study allows for both growth and decline to be studied and is detailed in the following section. The contribution of each measurement to brain development was further assessed with multiple linear regression of all the measurements of the same type (e.g. volume, surface area) against the age at scan.

The effect of preterm birth was assessed by comparing the group of term controls with an equal-sized group of early preterm subjects, born before 30 weeks, with equivalent ages at scan. Only a single scan per subject was included in the group analysis. There was no significant difference at the age at scan of the two groups (unpaired *t*-test, *p* = 0.98), however 10 of the preterm subjects had chronic lung disease. Group comparison was performed with unpaired t-tests when the data were normally distributed. Data that did not approximate a normal distribution were log transformed and checked again for normality. If the data were normally distributed in the log domain, comparison was again performed with an unpaired *t*-test in the log transformation. Non-normal distributions were compared with the non-parametric Wilcoxon rank sum test. Normality was assessed with the Lilliefor's test. Due to the large number of regions (82 in total) and the small number of term controls used in this study, we assess the effect of preterm birth using only univariate statistics.

The impact of increasing prematurity was further explored for all the preterm subjects by assessing correlations of the volume and surface measurements with GA at birth, correcting for the PMA at scan (correlation coefficient and adjusted *R*^2^ values). The measurements were further entered in a multiple regression model with GA at birth (correcting for the PMA at scan) to explore their relative importance in a combined model.

We assessed the effect of including multiple scans of preterm infants in the analysis with a linear mixed-effects model. Linear mixed-effects have been used in the literature for analysis of longitudinal data (Bernal-Rusiel et al., 2013, Sadeghi et al., 2013). We examined the relationship with brain development and increasing prematurity by entering the age at scan and age at birth (correcting for age at scan) respectively as fixed effects into the model. In order to account for the correlation of measurements within the same subject, we included intercepts for the subjects as random effects. After fitting the model, we examined the significance of the random effect with a likelihood ratio test of the linear mixed model and a linear model (the equivalent of the linear mixed model without the random effect). Marginal *R*^2^ values (Nakagawa and Schielzeth, 2013) of the fixed effects are reported and compared with the adjusted *R*^2^ values from the linear model.

Univariate statistics in all cases were adjusted with Bonferroni correction. Significance was assumed at *p* < 0.05. Table 7, Table 8 present a summary of the statistically significant measurements in this study based on the univariate and multivariate statistics respectively. Tables presenting all the results of the analysis in this study can be found in the supplementary material.

### Gompertz model

**Fig. 11:**
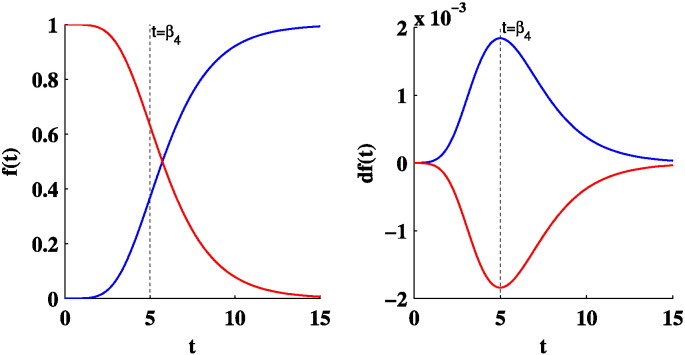
Example of a positive Gompertz function (blue line) that models growth and a negative Gompertz function (red line) that models decline as a function of time *t*. The left plot displays the function *f*(*t*) and the right plot the gradient of the function *df*(*t*). The peak growth/decline occurs at time *t* = *β*_*t*_ displayed with a dotted line.

Gompertz functions are sigmoid functions often used to model growth, where growth is initially low, then accelerates to reach peak growth, and then decelerates to low growth again at the end (see Fig. 11). As in Wright et al. (2014), we examine the following Gompertz-like function to model the relation of brain descriptors with age:

$$f\left(t\right)=\beta_{1}+\beta_{2}e^{-e^{-\beta_{3}\left(t-\beta_{4}\right)}}$$

where *β*_1_ is the initial value of *f*(*t*) and *β*_1_ + *β*_2_ the ending value of *f*(*t*). *β*_3_ is the parameter that adjusts the growth rate and *β*_4_ the *t* value where *f*(*t*) reaches peak growth. In this study we deviate from Wright et al. (2014) by allowing the parameters of the Gompertz function to take negative values. This allows us to further model decline of a descriptor in relation to age (see Fig. 11).

## Results

### Volumetric measurements

**Fig. B.1:**
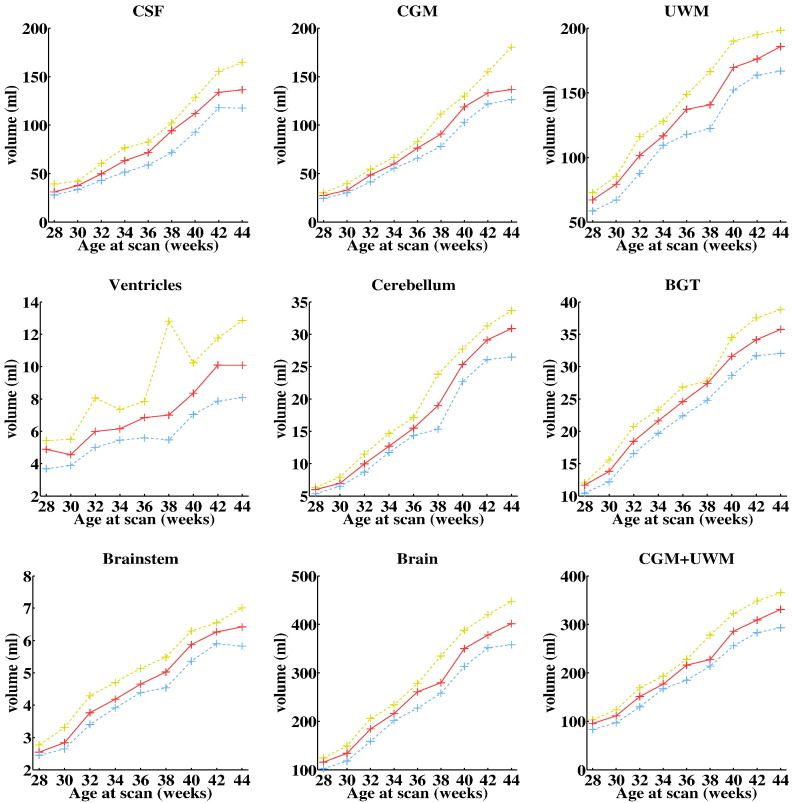
Volume(mL) centiles of tissues with increasing scan age. The yellow line for each region represents the 25% centile over the subjects in the database, the red line the median value, and the blue line the 75% centile.

**Fig. B.2:**
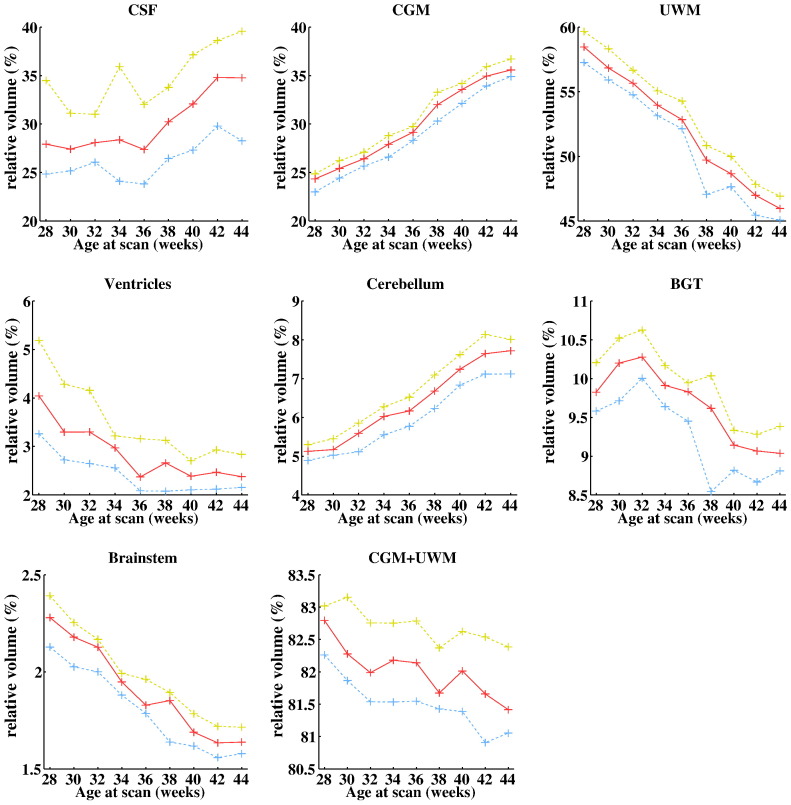
Relative volume (ratio of structure's volume to the total brain volume) centiles of tissues with increasing scan age. The yellow line for each region represents the 25% centile over the subjects in the database, the red line the median value, and the blue line the 75% centile.

**Fig. B.3:**
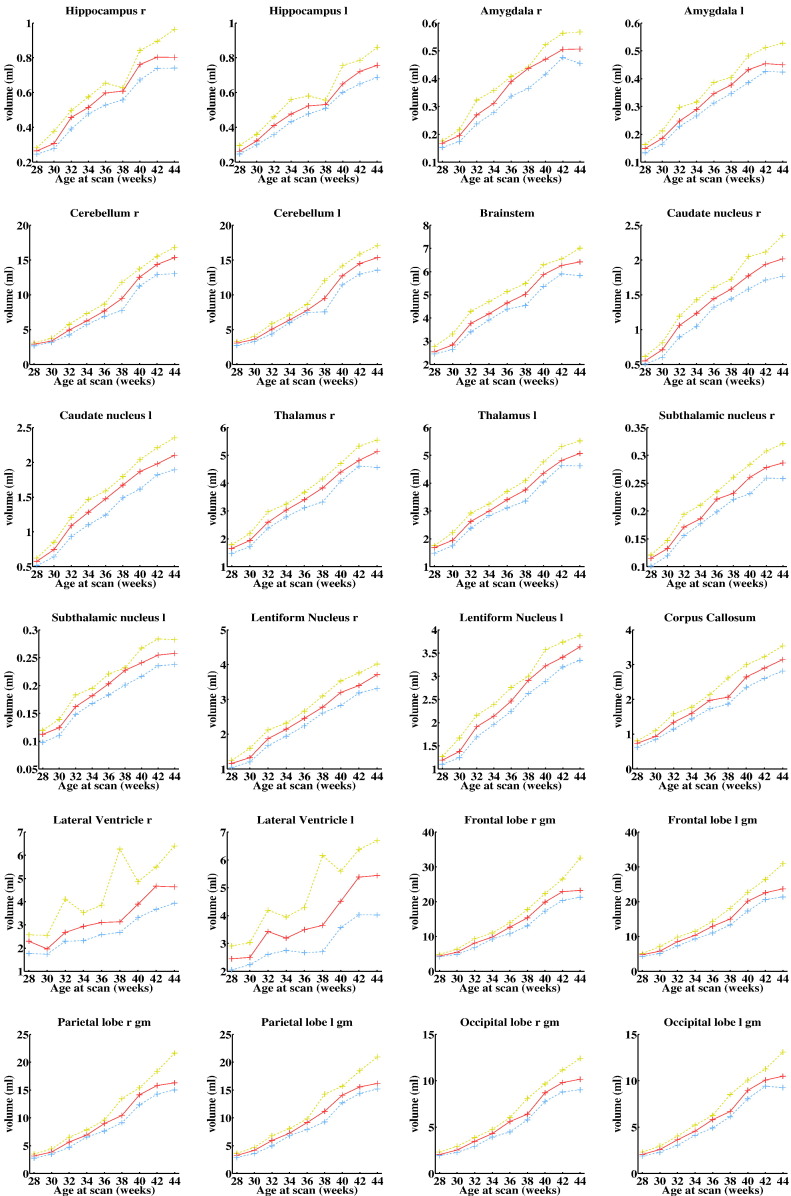

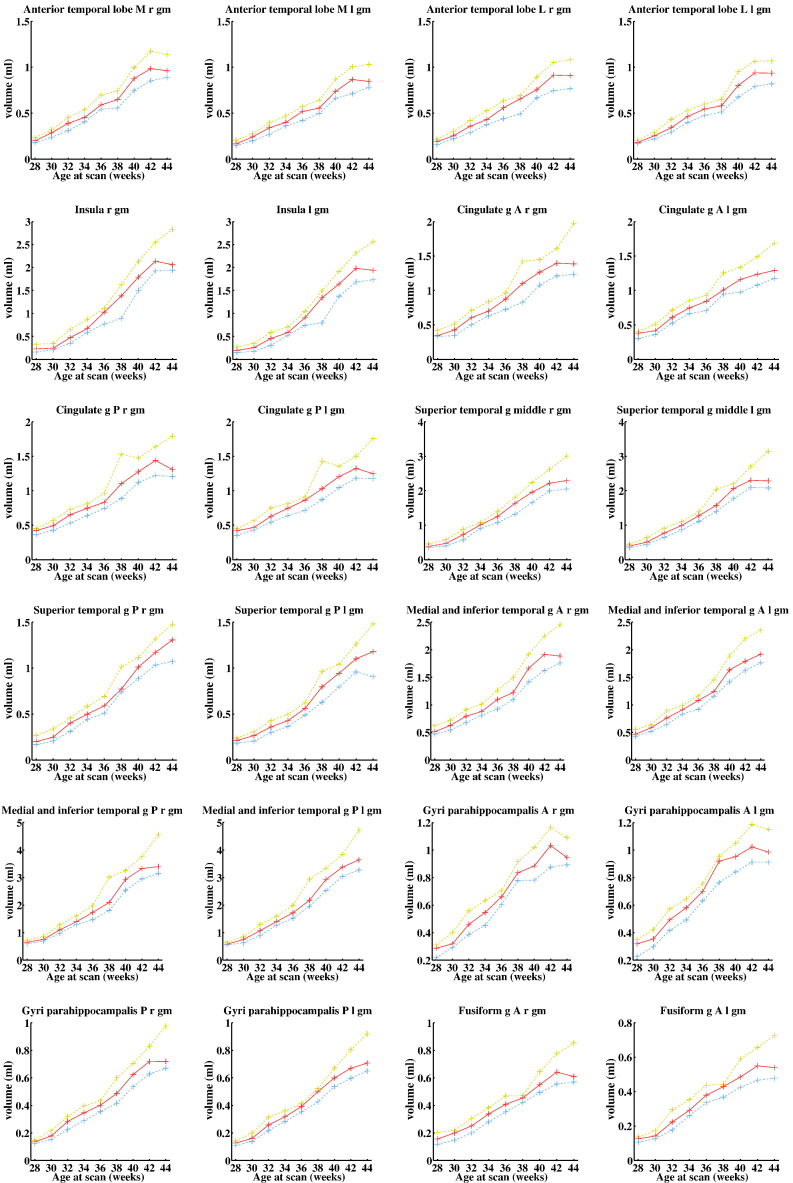

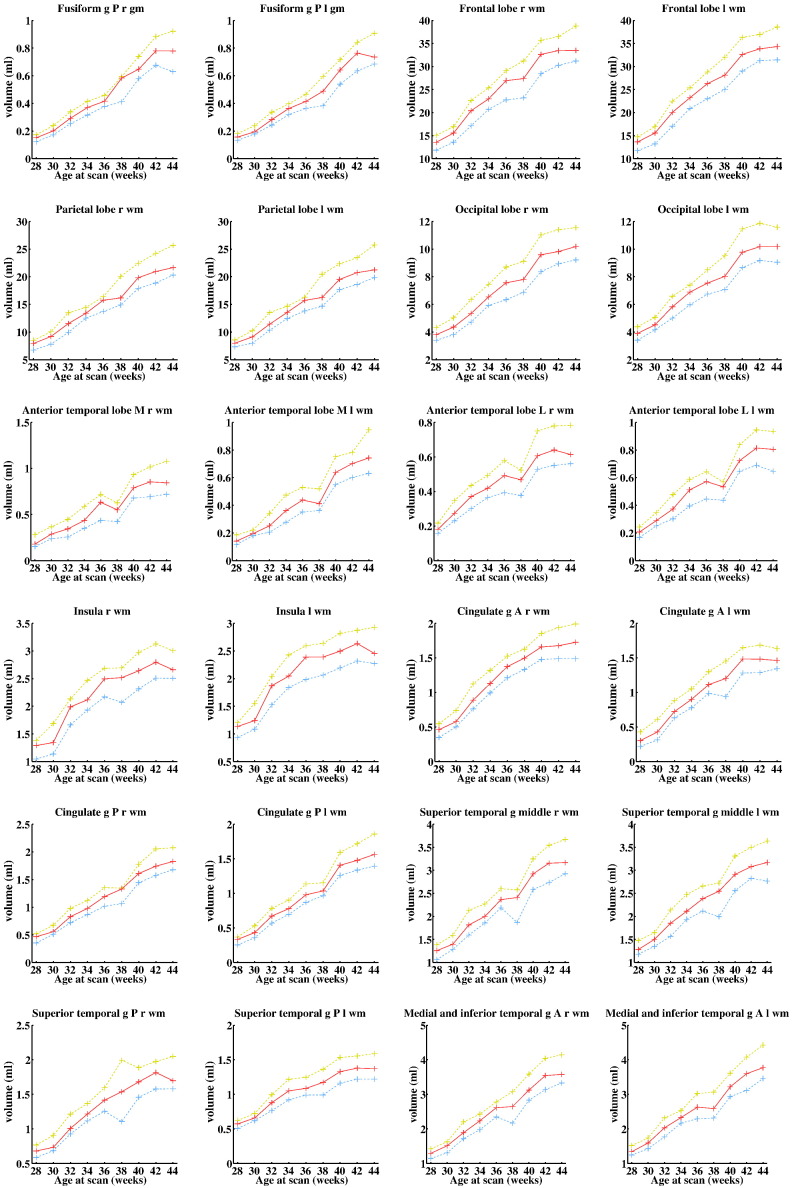

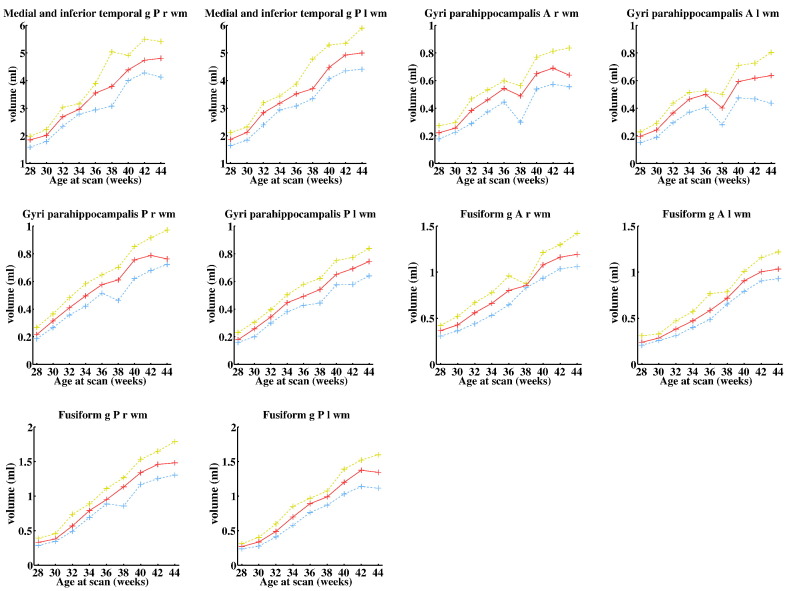
Volume(mL) centiles of the 82 structures of the whole brain with increasing scan age. The yellow line for each region represents the 25% centile over the subjects in the database, the red line the median value, and the blue line the 75% centile.

**Fig. 12:**
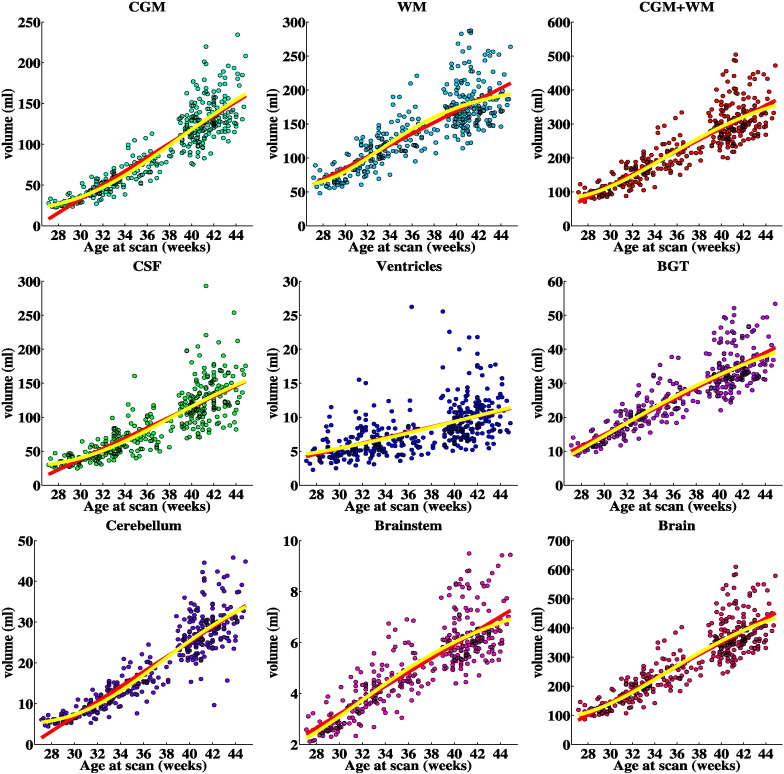
Tissue volumes of the preterm infants with increasing PMA at scan. The red line represents the linear regression fit and the yellow line the Gompertz fit to the data.

**Fig. 13:**
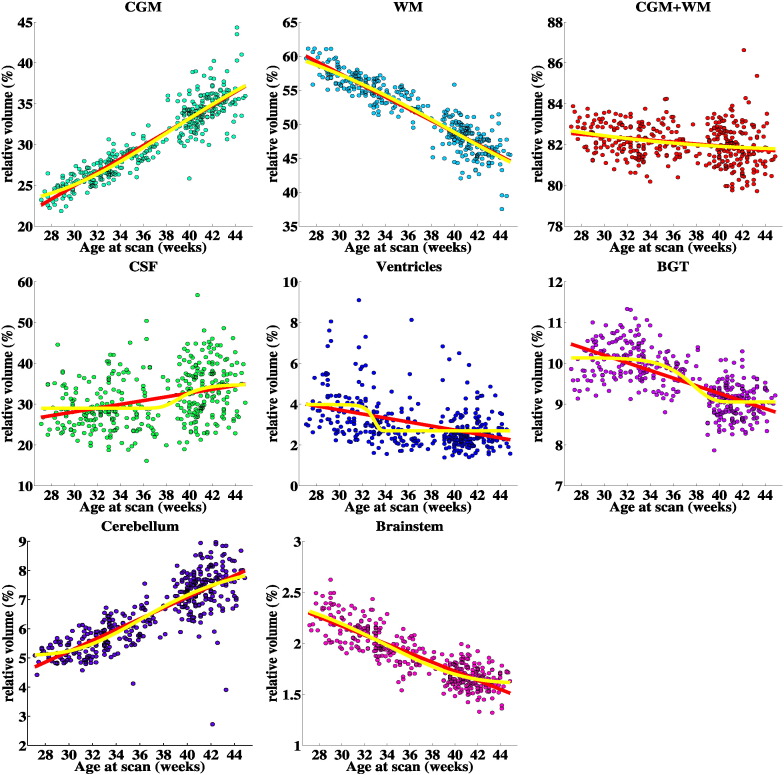
Relative tissue volumes of the preterm infants with increasing PMA at scan (% of the total brain volume). The red line represents the linear regression fit and the yellow line the Gompertz fit to the data.

Absolute and relative volumes of the brain tissues with increasing age at scan are illustrated in Fig. 12, Fig. 13 and centiles in Fig. B.1, Fig. B.2 of Appendix B respectively. Absolute volumes for all the 82 structures are presented in Fig. B.3 of Appendix B.

The absolute volume of each of the 82 structures was significantly correlated with the age at scan. In addition, most of the structures have a significant linear correlation of their relative volumes to age at scan. These correlations were positive for relative volume of the CGM, CSF, cerebellum and corpus callosum and negative for relative volume of the ventricles, the majority of WM regions and the basal ganglia and thalami. The Gompertz model fitted more accurately the relation of both absolute and relative volumetric measurements with age at scan for the majority of regions. However, the reduction in the sum of squares error was smaller than 5% for most of the measurements. This suggests a significant linearity in the structure development. The structures that their volume was better fitted with the Gompertz function were: the anterior cingulate gyrus WM bilaterally, insula WM and CGM bilaterally, posterior medial and inferior temporal gyrus CGM bilaterally and the left posterior superior temporal gyrus WM, CGM. The WM and the WM parts reach a peak growth at around 33 weeks age at scan, while the CSF and CGM and its parcellations present peak growth later at around 38–39 weeks. The subcortical structures have the largest growth around the 31 weeks, with the exception of cerebellum and ventricles that present the largest increase in volume later at 37 and 40 weeks respectively. The total brain growth reaches peak growth at the age of 35 weeks. When entered in a multiple regression model with age at scan, the structures that had significant effect on the model were: brainstem, corpus callosum, cerebellum right, subthalamic nucleus left, the WM and CGM parts of the left medial anterior temporal lobe and left anterior fusiform gyrus, and the WM parts of right anterior cingulate gyrus, left posterior superior temporal gyrus, anterior gyri parahippocampalis bilaterally. The combined model was highly correlated with age at scan with adjusted *R*^2^ = 0.935.

Total brain volume of the preterm group was significantly smaller than the term controls. Reduction in total brain volume was significantly associated with increasing prematurity in the preterm population.

The preterm infants had significantly reduced WM volume and more specifically in the lateral anterior temporal lobe bilaterally, anterior medial and inferior temporal gyrus bilaterally and the left hemispheric parts of the parietal lobe, medial anterior temporal lobe, posterior cingulate gyrus, middle superior temporal gyrus and anterior gyri parahippocampalis. Increasing prematurity was associated with a reduction in total and regional WM volume in the majority of WM parts.

The CGM was less affected overall with significant group differences localised in the lateral anterior temporal lobe bilaterally and the left medial anterior temporal lobe. Decreasing GA at birth was significantly associated with reduced total CGM volume and was negatively correlated with CGM volume in the medial and lateral anterior temporal lobe bilaterally, medial and inferior temporal gyrus bilaterally, anterior gyri parahippocampalis bilaterally, anterior fusiform gyrus bilaterally left frontal lobe, left anterior cingulate gyrus and the right middle superior temporal gyrus.

The volume of subcortical structures was also affected by prematurity. The preterm subjects had reduced volume in the areas of the corpus callosum, subthalamic nucleus, left amygdala and right caudate nucleus. Volumes of all the subcortical structures were significantly reduced with increasing prematurity. The absolute volume of the left ventricle and relative volume of CSF and both ventricles were significantly increased for the preterm subjects. This increase of relative volume was additionally correlated with increasing prematurity. Prematurity was further associated with relative volume changes in multiple brain regions.

The tissues that were significantly associated with increasing prematurity according to multiple linear regressions were the WM, brainstem, CSF and ventricles. The multiple regression model including all the 82 structures resulted in the following significant predictors of increasing prematurity: the WM parts of left medial anterior temporal lobe, right lateral anterior temporal lobe, left posterior cingulate gyrus, left anterior and right posterior medial and inferior temporal gyrus, left anterior fusiform gyrus and the anterior gyri parahippocampalis, the CGM parts of left frontal lobe, right occipital lobe, right anterior cingulate gyrus, right middle superior temporal gyrus, left anterior and posterior gyri parahippocampalis, and the brainstem, corpus callosum and left lateral ventricle.

Accounting for the repeated measurements with the linear mixed model yields statistically different results from the linear model as assessed by the likelihood ratio. However, the obtained *R*^2^ values are very similar to the linear model's *R*^2^ values. The vast majority of *R*^2^ values (98%) computed to quantify brain development and effect of prematurity had difference between the 2 models less than 0.02, which can be considered negligible.

### Cortical surface measurements

**Fig. 14:**
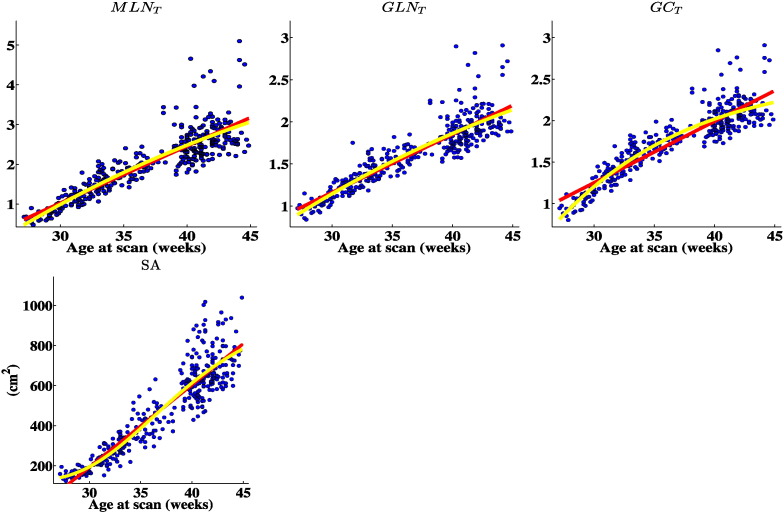
Cortical surface measurements of the preterm infants with increasing age at scan. The red line represents the linear regression fit and the yellow line the Gompertz fit to the data.

The different surface measures of the cortex with respect to age at scan are illustrated in Fig. 14 and correlations in Table 4. An initial experiment was performed to look into the effect of the Laplacian smoothing of the cortical surfaces. Cortical surfaces reconstructed with and without Laplacian smoothing present very similar results in correlation with ages at birth and scan. When compared with an unpaired *t*-test, the mean curvature *L*^2^ norm and global curvedness were significantly different between the two reconstructions, while the surface area and Gaussian curvature *L*^2^ norm did not reach significance. In the following, we present the analysis based on the smoothed surfaces.

The curvature measures and surface area were positively related to the PMA at scan for the whole cortex and for almost all the cortical regions after Bonferroni correction (with the exception of the mean curvature *L*^2^ norm and global curvedness of the left anterior gyri parahippocampalis). The regions whose surface area was significantly related to age at scan in a multivariate model were: right parietal lobe, left occipital lobe, right anterior cingulate gyrus, middle superior temporal gyrus bilaterally, anterior medial and inferior temporal gyrus bilaterally, left anterior and right posterior fusiform gyrus. Similarly for the curvature measures, the common regions that presented significant associations were: left medial anterior temporal lobe, left posterior superior temporal gyrus, anterior medial and inferior temporal gyrus bilaterally and left anterior gyri parahippocampalis. The adjusted *R*^2^ values of the multivariate model for surface area, mean curvature *L*^2^ norm, Gaussian curvature *L*^2^ norm and global curvedness were respectively: *R*^2^ = 0.896, *R*^2^ = 0.921, *R*^2^ = 0.906, *R*^2^ = 0.916. The relative surface area, the surface area of a region normalized to the total surface area, presents both regional increases and decreases with increasing age at scan that are significantly associated in the majority of cortical regions.

The Gompertz model provided a better approximation to the surface measures with respect to age at scan than the linear model in the majority of regions. However, in all the measures apart from the global curvedness the improvement in the SSE for most regions was not considerable (less than 5%). Exception is the surface area of the anterior gyri parahippocampalis and the curvatures of the posterior parts of the medial and inferior temporal gyrus and fusiform gyrus, and the medial and lateral parts of the anterior temporal lobe. The global curvedness of more than half the cortical regions presented an improvement in SSE larger than 5% with decreases up to 31%. The structures that presented the largest gain from the Gompertz fitting were the posterior part of the medial and inferior temporal gyrus and the frontal, parietal and occipital lobe. The increase in surface area is maximised at the ages (at scan) of 34–38 weeks. Most of the regional curvature measures present peak growth at either the earliest or latest ages at scan.

The cortical surface area was found to be significantly reduced in the preterm subjects and more specifically in the lateral anterior temporal lobe bilaterally and the left hemispheric parts of the medial anterior temporal lobe and anterior gyri parahippocampalis. Increasing prematurity was further associated with decreasing surface area in the whole cortex and most of the regions. From a multiple linear regression model, the structures that were significantly related to the age at scan were: right frontal lobe, right parietal lobe, right occipital lobe, left medial anterior temporal lobe, right anterior temporal lobe, right middle superior temporal gyrus, left anterior medial and inferior temporal gyrus, posterior medial and inferior temporal bilaterally and left anterior gyri parahippocampalis.

The curvature measurements were not associated with age at birth in the whole cortex and the majority of cortical regions. A notable exception is the anterior part of the temporal lobe that consistently presented a positive correlation with increasing prematurity in all of the curvature measures. The preterm infants further demonstrated increased curvature measurements compared to the term controls, especially in the mean curvature *L*^2^ norm and global curvedness. The right parts of the parietal lobe, posterior medial and inferior temporal gyrus, posterior superior temporal gyrus were significantly higher in the preterm subjects in all the curvature measures. These structures additionally presented increased relative volume in the preterm subjects and this increase was correlated with increasing prematurity. Increasing prematurity was significantly associated according to a multiple regression model with: the right occipital lobe, right anterior medial and inferior temporal gyrus, left anterior and right posterior gyri parahippocampalis and left anterior and right posterior fusiform gyrus.

Similar to the volumetric results, *R*^2^ values computed with the linear mixed model were essentially the same as that of the linear model with a maximum difference of 0.014 across all the surface measurements with respect to age at scan and age at birth.

### Comparison with manual measurements

The tissue volumes obtained after the proposed corrections are similar to volumes in the literature evaluated using manual segmentation approaches. Anbeek et al. (2008) provide average tissue volumes (mL) of 13 subjects around term who were born over a wide age range of gestations (gestational age 25.9–42.9 weeks, corrected age at test -3.6–5.1 weeks): CSF 51.4, CGM 101.2, WM 146.4, BGT 20, Brain 319. Corresponding CGM and WM volumes are obtained here over the scan ages of 36–40 weeks (see Fig. B.3 of Appendix B). The CGM in Anbeek et al. (2008) represents about 32% of the brain volume and the WM around 46% of the brain volume. The relative volumes obtained here are 34% for the CGM and 48% for the WM around term (see Table 5). It should be noted that the relative volumes prior to correction for the CGM–WM partial volume and sulci correction were 43% and 40% for the CGM and WM respectively. This overestimation of CGM obtained prior to the corrections is consistent with previous automatic segmentation studies (see Table 6).

**Fig. 15:**
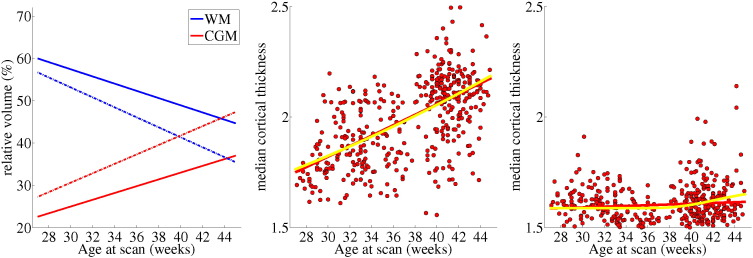
Relative WM and CGM volumes (left plot) and cortical thickness (centre and right plot) with increasing age at scan with and without the proposed CGM–WM partial volume correction and sulci correction. The left plot presents the volumetric results obtained by a Gaussian Mixture Model that assumes one class for WM and one class for CGM with dotted lines, and the results including the proposed corrections with continuous lines. The centre and right plot present the thickness results without and with the proposed corrections respectively. The red line in the thickness plots represents the linear regression fit and the yellow line the Gompertz fit to the data.

Similar volumes to manual results are further obtained for the early preterm period after the proposed corrections (see Table 5). Moeskops et al. (2013) present a relative CGM volume of 18% and relative WM volume of 70% for 10 neonates scanned at 30.8 ± 0.7 weeks age at scan. The relative volumes in our study are 25% for the CGM and 57% for the WM around 30 weeks age at scan (prior to the corrections the relative CGM and WM were 31% and 52% respectively). The CGM oversegmentation caused by a Gaussian Mixture Model that assumes one class for WM and one class for CGM can be observed in Fig. 15. Without the introduction of the CGM–WM partial volume correction, the segmentation tends to attribute a larger proportion of the brain to the CGM.

Median thickness across the subjects in the cohort is presented in Fig. 15. The cortical thickness estimated using the cortical segmentations without the sulci correction produces an increasing thickness with age at scan. The thickness of the uncorrected segmentations correlates significantly with the age at scan (*p* < 10^− 36^). However, with the introduction of the sulci correction, the cortical thickness measured over the subjects remains unaffected by the age at scan (*p* = 0.07) of the neonate. The cortical thickness of the neonatal brain has a median value of 1.59 ± 0.09 mm across the database (the 25th and 75th percentiles are 1.54 and 1.65 mm respectively). A Gompertz fit to the thickness data decreases the mean squared error by a factor of 2% and displays a gradual increase of thickness after the age of 38 weeks from 1.59 to 1.65 mm.

## Discussion

Quantitative measurements of the developing neonatal brain are required to study normal brain growth and potentially aid the early diagnosis of later neurological impairments. In this study, we employed the automatic segmentation algorithm proposed in Makropoulos et al. (2014) to delineate 82 regions of the brain in a large group of infants and derive a number of volumetric and cortical surface measurements. Two corrections are incorporated for the detailed delineation of the cortical ribbon in the neonatal brain. The first correction estimates a partial volume class between the CGM and WM which is consequently relabelled as WM in order to limit the over-inclusion of voxels in the CGM tissue. The second correction detects and delineates the cortical sulci that are hard to segment with intensity-based segmentation techniques. We initially detect the cortical sulci from the expansion of the interior cortical surface as areas of the surface that collapse to each other similar to Han et al. (2004). The thickness of the detected sulcal areas is then approximated from neighbouring parts of the cortical ribbon where the thickness can be accurately measured. It should be noted that the sulci correction proposed assumes a similar cortical thickness in the local neighbourhood of the cortical ribbon. This could potentially introduce errors in regions where this assumption does not hold. However since the cortical thickness is typically less than 2 voxels, we would expect only negligible errors from this assumption. Derived volumetric and thickness measures after the application of the method presented here are similar to measurements obtained from manually segmented data. A structural atlas is constructed for different ages of the neonatal brain for all the segmented brain structures and is made publicly available. The atlas defines the structure probability and average segmentation respectively of each structure in the spatio-temporal space of Serag et al. (2012).

Cortical surface area measurements have been previously presented for the neonatal brain with a range of 150–1500 cm^2^ between 27 and 44 weeks PMA at scan (Kapellou et al., 2006, Xue et al., 2007, Dubois et al., 2008b, Pienaar et al., 2008, Rodriguez-Carranza et al., 2008, Moeskops et al., 2013, Moeskops et al., 2015). The surface area of the cortex in this study was around 120–1100 cm^2^ in the corresponding ages at scan. Curvature measurements have been reported in a limited number of studies (Xue et al., 2007, Pienaar et al., 2008, Rodriguez-Carranza et al., 2008, Moeskops et al., 2013, Moeskops et al., 2015) which used different definitions of curvature measures and included only small numbers of subjects. Here, the curvature measures from Rodriguez-Carranza et al. (2008) were adopted that are invariant to the surface area. Similar positive correlations of cortical curvature with age at scan to Rodriguez-Carranza et al. (2008) are derived in this analysis. Xue et al. (2007), Dubois et al. (2008b), Moeskops et al. (2015) likewise presented increasing mean curvature and gyrification with increasing age at scan. We should note here that the cortical surfaces were smoothed prior to obtaining the surface measurements which might influence the results. Laplacian smoothing did not have a major impact on consequent measurements. The cortical surface was reconstructed after blurring the cortical mask to avoid a blocky appearance due to the limited resolution, which would lead to extreme curvature values. Although this might affect the measurements, we do not expect a significant effect due to the small amount of blurring introduced (Gaussian kernel of 1 mm standard deviation) and due to the fact that median values are only reported in this study.

Cortical thickness measurements in the neonatal population have been previously presented in (Xue et al., 2007, Moeskops et al., 2013, Moeskops et al., 2015) for limited datasets. These studies obtain a median cortical thickness of around 1–1.4 mm for the neonatal brain. Moeskops et al. (2015) further demonstrate an increase in cortical thickness from around 1 mm to 1.4 mm between 30 and 40 weeks age at scan in the preterm brain. The cortical thickness estimated here remains almost constant in the neonatal brain, similar to Xue et al. (2007), with a value around 1.6 mm, from the early preterm period to term-equivalent age. Differences in thickness values can be attributed to the different in-plane resolution of the MRI (Moeskops et al., 2013, Moeskops et al., 2015 have a highly anisotropic resolution, 0.34 × 0.34 × 2 and 0.35 × 0.35 × 1.2 mm, while the analysed data have a near isotropic resolution of 0.86 × 0.86 × 1 mm), and different thickness measurement methods (Xue et al., 2007).

We also obtained absolute and relative volumes of brain tissues from the early preterm period to term-equivalent age. Surface area and curvature measures of the whole cortex and regional cortical parts were estimated based on the segmentations. Our results show that, with the exception of cortical thickness, regional brain and cortical growth is significantly associated with brain maturation. A Gompertz function presents a better approximation than a linear model for the relation of the volumetric and surface measurements with age at scan. This is expected given the increased degrees of freedom of the Gompertz function (4 variables to adjust instead of 2 for the linear model). However, the reduction in the sum of squares error with respect to the linear model is less than 5% in the majority of measurements, suggesting that the linear model can still capture the relationship with age at scan. A similar relationship of cortical folding with age is exhibited in the fetal brain (Wright et al., 2014) over the ages of 27–39 weeks. However, the fetal data in Wright et al. (2014) additionally cover an earlier spectrum of ages starting from 22 weeks gestational age. Over the age range of 22–27 weeks the fetal brain presents large, non-linear, increases in cortical folding with peak growth at 30–32 weeks. Consequently, the Gompertz model in the fetal data provides a better approximation with a fitting error approximately half of the linear model (Wright et al., 2014). A recent study comparing cortical folding between preterm newborns and fetuses (Lefevre et al., 2015) presented similar results. The cortical curvature in both the fetal and neonatal brain demonstrated a linear relationship with age over the range of around 28–36 weeks, although of different extent in the two groups. The fetal brains prior to that age range demonstrated a non-linear increase with age, although this effect was not specifically explored (Lefevre et al., 2015). The neonatal brain presents peak volumetric growth at 35 weeks age at scan which is presented progressively at the subcortical regions except for the cerebellum, the WM regions, the cerebellum, the CSF, the CGM parts and the ventricles. The growth of the regional surface area is maximised at the ages of 34–38 weeks. Multivariate analysis of the volumetric and surface measures including all the regions result in adjusted *R*^2^ values ranging from 0.896 to 0.935.

We used the volumetric, surface area and curvature measures to characterise the effect of prematurity in the neonatal brain, and to compare preterm brain development with that of healthy term born controls. Total brain volume in the preterm infants was reduced compared to term controls. CLD has been previously associated with reduced total brain volume compared to preterm infants without CLD who do not seem to have reduced brain volumes (Boardman et al., 2007). Almost half the infants in the preterm group (17 out of 40) had CLD and this may be reflected in the reduced brain volume in this study. Total brain volume was also highly negatively correlated to increasing prematurity, as has been reported previously (Peterson et al., 2003, Inder et al., 2005, Thompson et al., 2007, Ball et al., 2012). Prematurity is related to reductions in the volume of the WM, CGM and subcortical structures, and increases in the relative CSF and ventricular volume and these alterations are significantly associated with decreasing age at birth. Regional decreases in the WM have been previously described in Mewes et al. (2006), and Thompson et al. (2007). These studies presented both reductions and increases in the regional CGM volumes. Similar volumetric associations in the subcortical GM and more specifically in the amygdala, thalamus, hippocampus and lentiform nucleus have been reported in Peterson et al. (2000), Srinivasan et al. (2007), and Ball et al. (2012). Larger volumes of CSF and ventricles in the preterm subjects have been found in previous studies (Peterson et al., 2000, Mewes et al., 2006, Thompson et al., 2007).

Preterm infants had reduced surface area compared to term controls in only a few parts of the cortex. Surface area reductions were however significantly correlated with increasing prematurity in the majority of the cortical regions. Ajayi-Obe et al. (2000) similarly presented reduced cortical surface area in preterm infants compared to term controls. Kapellou et al. (2006) further demonstrated a decreasing surface area in the cortex with increasing prematurity. Cortical curvature was largely not associated with the age at birth of the infants. An exception is the anterior temporal lobe that presents a positive correlation with increasing prematurity. Kesler et al. (2006) demonstrated similar results in prematurely-born children, where the temporal lobe was shown to be specifically disrupted by preterm delivery with increased gyrification in the preterm population. Kesler et al. (2006) suggested that increased gyrification may be due to abnormal growth of the inner cortical layers. Here, these alterations are specifically localised in the anterior part of the temporal lobe. The preterm infants further demonstrated increased curvature measurements compared to the term controls. Specifically, the curvature of the right parts of the parietal lobe, posterior medial and inferior temporal gyrus and posterior superior temporal gyrus was significantly higher in the preterm subjects for all the curvature measures. The relative volume of these structures was also significantly increased compared to term controls and was correlated with increasing prematurity. Future studies with the inclusion of clinical variables and neurodevelopmental outcome will help to further elucidate the effect of prematurity in the neonatal brain.

## Figures and Tables

**Table 1:** Cohort characteristics. Median (range) age, weight and head circumference at the time of birth and scan are presented. The number of preterm infants with chronic lung disease, patent ductus arteriosus and culture positive sepsis is reported.

	Preterm infants	Term controls
Number of infants/images:		
Total number	298/380	40/40
Chronic lung disease (CLD)	49/54	–
Patent ductus arteriosus (PDA)	32/38	–
Culture positive sepsis	23/28	–
Age at birth (weeks)	29^+ 3^ (23^+ 2^–36)	39^+ 2^ (36^+ 1^–42)
Age at scan (weeks)	37^+ 6^ (27^+ 1^–44^+ 6^)	40^+ 6^ (37–44^+ 4^)
Postnatal age at scan (weeks)	6^+ 5^ (0^+ 1^–19^+ 5^)	0^+ 6^ (0–5^+ 5^)
Weight at birth (kg)	1.17 (0.54–3.71)	3.42 (1.93–4.34)
Weight at scan (kg)	2.19 (0.64–5.5)	3.48 (1.93–4.71)
Head circumference at birth (cm)	27 (20–38.5)	34.3 (30.2–38.7)
Head circumference at scan (cm)	31.6 (22–39.6)	35.4 (30.2–38)

**Table 2:** Regional structures of the brain segmented using the automatic method in Makropoulos et al. (2014).

WM, CGM structures	Subcortical regions
Frontal lobe (left/right)	Hippocampus (left/right)
Parietal lobe (left/right)	Amygdala (left/right)
Occipital lobe (left/right)	Cerebellum (left/right)
Anterior temporal lobe, medial part (left/right)	Brainstem
Anterior temporal lobe, lateral part (left/right)	Caudate nucleus (left/right)
Gyri parahippocampalis et ambiens, anterior part (left/right)	Thalamus (left/right)
Gyri parahippocampalis et ambiens, posterior part (left/right)	Sub-thalamic nucleus (left/right)
Superior temporal gyrus, middle part (left/right)	Lentiform nucleus (left/right)
Superior temporal gyrus, posterior part (left/right)	Corpus callosum
Medial and inferior temporal gyrus, anterior part (left/right)	Lateral ventricles (left/right)
Medial and inferior temporal gyrus, posterior part (left/right)	
Fusiform gyrus, anterior part (left/right)	
Fusiform gyrus, posterior part (left/right)	
Insula (left/right)	
Cingulate gyrus, anterior part (left/right)	
Cingulate gyrus, posterior part (left/right)	

**Table 3:** Area-independent curvature measures. Notation: H = mean curvature, G = Gaussian curvature, c = curvedness, A = surface area, T = 3*volume/A.

Global curvedness	$GC_{T}=\frac{T}{A}\sum_{A}c$
Mean curvature *L*^2^ norm	$\mathrm{ML}N_{T}=\frac{T^{2}}{A}\sum_{A}H^{2}$
Gaussian curvature *L*^2^ norm	$\mathrm{GL}N_{T}=T\sqrt[{4}]{\frac{1}{A}\sum_{A}K^{2}}$

**Table 4:** Correlations of surface measures with PMA at scan, and GA at birth correcting for the PMA at scan (bold = significant at *p* < 0.05). Results for the cortical surfaces reconstructed with and without Laplacian smoothing are presented. The first two rows present the correlation coefficients and the next two rows the adjusted *R*^2^ values.

	After Laplacian smoothing				Without smoothing			
	*SA*	*MLN_T_*	*GLN_T_*	*GC_T_*	*SA*	*MLN_T_*	*GLN_T_*	*GC_T_*
PMA at scan	0.921	0.880	0.894	0.909	0.925	0.865	0.883	0.903
GA at birth | PMA at scan	0.244	− 0.039	− 0.017	0.089	0.242	− 0.048	− 0.029	0.075
PMA at scan	0.849	0.774	0.799	0.826	0.855	0.748	0.779	0.816
GA at birth | PMA at scan	0.057	− 0.001	− 0.002	0.005	0.056	0	− 0.002	0.003

**Table 5:** Relative CGM and WM volumes in the early preterm (study A:Moeskops et al., 2013) and term period (study B:Anbeek et al., 2008) reported using manual segmentation and the proposed technique with and without correction for the CGM–WM partial volume and sulci correction.

Study	Early preterm period	Term period
A, B	CGM: 18%, WM: 70%	CGM: 32%, WM: 46%
Proposed, no corrections	CGM: 31%, WM: 52%	CGM: 43%, WM: 40%
Proposed	CGM: 25%, WM: 57%	CGM: 34%, WM: 48%

**Table 6:** CGM volumes (mL) around term-equivalent age reported with different automatic segmentation techniques (study A:Inder et al., 2005, study B:Thompson et al., 2007, study C:Cardoso et al., 2013) (mean ± standard deviation, centiles: 25%–75%).

Study	Preterm period	Term period
A	178 ± 41	227 ± 26
B	159 ± 41	173 ± 32
C	Centiles: 120–200	
Proposed, no corrections	164 ± 42, centiles: 139–184	176 ± 41, centiles: 144–193
Proposed	126 ± 31, centiles: 108–140	130 ± 30, centiles: 107–144

**Table 7:** Statistically significant regional measurements (noted with ✓) associated with brain development, preterm birth and increasing prematurity assessed with univariate statistics.

	Brain development							Preterm birth							Increasing prematurity						
	Volume	Rel. volume	SA	rel. SA	*MLN_T_*	*GLN_T_*	*GC_T_*	Volume	Rel. volume	SA	rel. SA	*MLN_T_*	*GLN_T_*	*GC_T_*	Volume	Rel. volume	SA	rel. SA	*MLN_T_*	*GLN_T_*	*GC_T_*
Hippocampus r	✓	✓													✓						
Hippocampus l	✓	✓													✓						
Amygdala r	✓	✓						✓							✓						
Amygdala l	✓	✓													✓						
Cerebellum r	✓	✓													✓						
Cerebellum l	✓	✓													✓						
Brainstem	✓	✓													✓						
Caudate nucleus r	✓	✓						✓							✓	✓					
Caudate nucleus l	✓	✓													✓	✓					
Thalamus r	✓	✓													✓						
Thalamus l	✓	✓													✓						
Subthalamic nucleus r	✓	✓						✓							✓						
Subthalamic nucleus l	✓	✓						✓							✓						
Lentiform nucleus r	✓	✓													✓						
Lentiform nucleus l	✓	✓													✓						
Corpus callosum	✓	✓						✓	✓						✓						
Lateral ventricle r	✓	✓							✓							✓					
Lateral ventricle l	✓	✓						✓	✓							✓					
Frontal lobe r gm	✓	✓	✓	✓	✓	✓	✓										✓				
Frontal lobe l gm	✓	✓	✓	✓	✓	✓	✓								✓		✓				
Parietal lobe r gm	✓	✓	✓	✓	✓	✓	✓		✓			✓	✓	✓		✓	✓				
Parietal lobe l gm	✓	✓	✓	✓	✓	✓	✓					✓		✓			✓				
Occipital lobe r gm	✓	✓	✓		✓	✓	✓			✓						✓	✓				✓
Occipital lobe l gm	✓	✓	✓	✓	✓	✓	✓			✓						✓	✓				
Anterior temporal lobe M r gm	✓	✓	✓		✓	✓	✓								✓		✓		✓	✓	✓
Anterior temporal lobe M l gm	✓	✓	✓		✓	✓	✓	✓		✓					✓		✓		✓	✓	✓
Anterior temporal lobe L r gm	✓	✓	✓	✓	✓	✓	✓	✓	✓	✓					✓	✓	✓		✓	✓	✓
Anterior temporal lobe L l gm	✓	✓	✓	✓	✓	✓	✓	✓	✓	✓	✓				✓	✓	✓		✓	✓	✓
Insula r gm	✓	✓	✓	✓	✓	✓	✓										✓				
Insula l gm	✓	✓	✓	✓	✓	✓	✓										✓		✓		
Cingulate g A r gm	✓	✓	✓	✓	✓	✓	✓		✓		✓	✓		✓		✓	✓				
Cingulate g A l gm	✓		✓	✓	✓	✓	✓				✓	✓		✓	✓		✓				
Cingulate g P r gm	✓	✓	✓	✓	✓	✓	✓		✓		✓					✓	✓				
Cingulate g P l gm	✓		✓	✓	✓	✓	✓		✓							✓	✓				
Superior temporal g middle r gm	✓	✓	✓		✓	✓	✓								✓	✓	✓	✓			
Superior temporal g middle l gm	✓	✓	✓	✓	✓	✓	✓										✓				
Superior temporal g P r gm	✓	✓	✓	✓	✓	✓	✓		✓		✓	✓	✓	✓		✓					
Superior temporal g P l gm	✓	✓	✓	✓	✓	✓	✓		✓			✓				✓	✓				
Medial and inferior temporal g A r gm	✓	✓	✓	✓	✓	✓	✓								✓		✓		✓		
Medial and inferior temporal g A l gm	✓	✓	✓	✓	✓	✓	✓					✓		✓	✓		✓				
Medial and inferior temporal g P r gm	✓	✓	✓	✓	✓	✓	✓		✓			✓	✓	✓		✓		✓			
Medial and inferior temporal g P l gm	✓	✓	✓	✓	✓	✓	✓					✓		✓		✓		✓			✓
Gyri parahippocampalis A r gm	✓		✓	✓	✓	✓	✓					✓			✓		✓				
Gyri parahippocampalis A l gm	✓		✓	✓		✓				✓		✓		✓	✓		✓				
Gyri parahippocampalis P r gm	✓	✓	✓	✓	✓	✓	✓		✓			✓					✓	✓			
Gyri parahippocampalis P l gm	✓	✓	✓		✓	✓	✓					✓					✓				
Fusiform g A r gm	✓	✓	✓	✓	✓	✓	✓								✓	✓	✓				
Fusiform g A l gm	✓	✓	✓		✓	✓	✓			✓					✓	✓				✓	✓
Fusiform g P r gm	✓	✓	✓	✓	✓	✓	✓				✓			✓							
Fusiform g P l gm	✓	✓	✓	✓	✓	✓	✓														
Frontal lobe r wm	✓	✓													✓	✓					
Frontal lobe l wm	✓	✓													✓	✓					
Parietal lobe r wm	✓	✓													✓						
Parietal lobe l wm	✓	✓						✓	✓						✓						
Occipital lobe r wm	✓	✓													✓						
Occipital lobe l wm	✓	✓													✓						
Anterior temporal lobe M r wm	✓	✓													✓						
Anterior temporal lobe M l wm	✓	✓						✓							✓						
Anterior temporal lobe L r wm	✓	✓						✓	✓						✓	✓					
Anterior temporal lobe L l wm	✓							✓	✓						✓	✓					
Insula r wm	✓	✓													✓						
Insula l wm	✓	✓													✓	✓					
Cingulate g A r wm	✓	✓													✓	✓					
Cingulate g A l wm	✓	✓													✓	✓					
Cingulate g P r wm	✓	✓													✓						
Cingulate g P l wm	✓	✓						✓	✓						✓						
Superior temporal g middle r wm	✓	✓													✓						
Superior temporal g middle l wm	✓	✓						✓							✓						
Superior temporal g P r wm	✓	✓													✓						
Superior temporal g P l wm	✓	✓													✓						
Medial and inferior temporal g A r wm	✓	✓																			
Medial and inferior temporal g A l wm	✓	✓						✓	✓						✓	✓					
Medial and inferior temporal g P r wm	✓	✓													✓	✓					
Medial and inferior temporal g P l wm	✓	✓													✓	✓					
Gyri parahippocampalis A r wm	✓	✓																			
Gyri parahippocampalis A l wm	✓	✓						✓							✓						
Gyri parahippocampalis P r wm	✓	✓													✓						
Gyri parahippocampalis P l wm	✓														✓						
Fusiform g A r wm	✓															✓					
Fusiform g A l wm	✓	✓														✓					
Fusiform g P r wm	✓	✓													✓						
Fusiform g P l wm	✓	✓													✓						

**Table 8:** Statistically significant regional measurements (noted with ✓) associated with brain development and increasing prematurity assessed with multivariate statistics.

	Brain development							Increasing prematurity						
	Volume	Rel. volume	SA	rel. SA	*MLN_T_*	*GLN_T_*	*GC_T_*	Volume	Rel. volume	SA	rel. SA	*MLN_T_*	*GLN_T_*	*GC_T_*
Hippocampus r		✓												
Hippocampus l														
Amygdala r														
Amygdala l														
Cerebellum r	✓	✓												
Cerebellum l														
Brainstem	✓	✓						✓						
Caudate nucleus r														
Caudate nucleus l														
Thalamus r														
Thalamus l		✓												
Subthalamic nucleus r														
Subthalamic nucleus l	✓	✓												
Lentiform nucleus r														
Lentiform nucleus l														
Corpus callosum	✓	✓						✓	✓					
Lateral ventricle r														
Lateral ventricle l								✓	✓					
Frontal lobe r gm										✓				
Frontal lobe l gm		✓			✓		✓	✓						
Parietal lobe r gm			✓							✓				
Parietal lobe l gm						✓	✓							
Occipital lobe r gm								✓		✓		✓	✓	✓
Occipital lobe l gm			✓											
Anterior temporal lobe M r gm							✓					✓		
Anterior temporal lobe M l gm	✓				✓	✓	✓			✓				
Anterior temporal lobe L r gm		✓								✓			✓	
Anterior temporal lobe L l gm													✓	
Insula r gm														
Insula l gm														
Cingulate g A r gm			✓					✓	✓				✓	
Cingulate g A l gm														
Cingulate g P r gm					✓									
Cingulate g P l gm						✓							✓	
Superior temporal g middle r gm			✓					✓	✓	✓				
Superior temporal g middle l gm			✓											
Superior temporal g P r gm					✓									
Superior temporal g P l gm					✓	✓	✓					✓		
Medial and inferior temporal g A r gm			✓		✓	✓	✓					✓	✓	✓
Medial and inferior temporal g A l gm					✓	✓	✓			✓				
Medial and inferior temporal g P r gm										✓				
Medial and inferior temporal g P l gm		✓	✓							✓			✓	
Gyri parahippocampalis A r gm							✓					✓		
Gyri parahippocampalis A l gm					✓	✓	✓	✓	✓	✓		✓	✓	✓
Gyri parahippocampalis P r gm												✓	✓	✓
Gyri parahippocampalis P l gm		✓						✓						
Fusiform g A r gm													✓	
Fusiform g A l gm	✓	✓	✓									✓	✓	✓
Fusiform g P r gm			✓				✓					✓	✓	✓
Fusiform g P l gm														
Frontal lobe r wm														
Frontal lobe l wm														
Parietal lobe r wm														
Parietal lobe l wm		✓												
Occipital lobe r wm														
Occipital lobe l wm														
Anterior temporal lobe M r wm														
Anterior temporal lobe M l wm	✓							✓	✓					
Anterior temporal lobe L r wm								✓						
Anterior temporal lobe L l wm														
Insula r wm														
Insula l wm														
Cingulate g A r wm	✓													
Cingulate g A l wm									✓					
Cingulate g P r wm														
Cingulate g P l wm								✓						
Superior temporal g middle r wm														
Superior temporal g middle l wm														
Superior temporal g P r wm														
Superior temporal g P l wm	✓													
Medial and inferior temporal g A r wm														
Medial and inferior temporal g A l wm								✓	✓					
Medial and inferior temporal g P r wm								✓						
Medial and inferior temporal g P l wm														
Gyri parahippocampalis A r wm	✓							✓						
Gyri parahippocampalis A l wm	✓							✓	✓					
Gyri parahippocampalis P r wm														
Gyri parahippocampalis P l wm														
Fusiform g A r wm														
Fusiform g A l wm	✓	✓						✓						
Fusiform g P r wm														
Fusiform g P l wm														

## Appendix A Sulci correction

‘Shock’ points are detected as in Han et al. (2004): The CGM–WM interface is propagated with the fast marching method. The distance *D*_*FMM*_ to the CGM–WM interface is defined by solving the following Eikonal equation:

$$F\left(i\right)∥\nabla D_{FMM}\left(i\right)∥=1$$

where *i* indexes the voxels of the image. *D*_*FMM*_(*i*) = 0 for voxels in the CGM–WM interface and *F*(*i*) = 1 − 0.9*p*_*i*,*CSF*_ according to the CSF posterior *p*_*CSF*_ of the EM algorithm. The gradient ∇*D*_*FMM*_ can subsequently be used to identify ‘shock’ points where the spatial derivative is not well defined:

$$S=\left\{i|F\left(i\right)∥\nabla D_{FMM}\left(i\right)∥<=T\right\}\;\text{.}$$

Here *T* is set to 0.8 as in Han et al. (2004). At these points two sulcal banks will merge in the propagated surface and ∇*D*_*FMM*_ will be small.

Sulcal points are then selected among the ‘shock’ points based on the assumption that the thickness of the cortical ribbon is locally consistent. A distance *D*_*allowed*,*i*_ is measured for each ‘shock’ voxel as the locally weighted mean cortical thickness measured on other points of the cortical ribbon. *D*_*allowed*,*i*_ is only averaged over points with streamlines that do not cross a ‘shock’ point, in essence points that are not inside a cortical sulcus. Connected components of ‘shock’ points, points that belong to the same sulcus, are identified with connected component labelling. Points that belong to the same sulcus are then defined to have the same *D*_*allowed*_, which is the mean *D*_*allowed*,*i*_ of the ‘shock’ points in the component. Finally, ‘shock’ points are labelled as CSF only if their distance to the CGM–WM interface *D*_*WM*,*i*_ is larger than *D*_*allowed*_.

## Appendix C Supplementary data

Volume centiles of the tissues and 82 structures of the brain with increasing scan age.

Correlations and adjusted R^2^ values of curvature measurements with age at birth correcting for the age at scan.

Correlations and adjusted R^2^ values of curvature measurements with age at scan.

Comparison of curvature measurements between the group of early preterm infants and the term controls.

Relative volume centiles of the tissues and 82 structures of the brain with increasing scan age.

Comparison of surface area between the group of early preterm infants and the term controls.

Surface area centiles of the 32 CGM structures of the brain with increasing scan age.

Correlations and adjusted R^2^ values of surface area with age at birth correcting for the age at scan.

Correlations and adjusted R^2^ values of surface area with age at scan.

Correlations and adjusted R^2^ values of absolute volumes with age at birth correcting for the age at scan.

Correlations and adjusted R^2^ values of absolute volumes with age at scan.

Comparison of volumes between the group of early preterm infants and the term controls.

## References

[bb0005] Ajayi-Obe M., Saeed N., Cowan F.M., Rutherford M.A., Edwards A.D. (2000). Reduced development of cerebral cortex in extremely preterm infants. Lancet.

[bb0010] Anbeek P., Vincken K.L., Groenendaal F., Koeman A., van Osch M.J.P., van der Grond J. (2008). Probabilistic brain tissue segmentation in neonatal magnetic resonance imaging. Pediatr. Res..

[bb0015] Ball G., Boardman J.P., Rueckert D., Aljabar P., Arichi T., Merchant N., Gousias I.S., Edwards A.D., Counsell S.J. (2012). The effect of preterm birth on thalamic and cortical development. Cereb. Cortex.

[bb0020] Bernal-Rusiel J.L., Reuter M., Greve D.N., Fischl B., Sabuncu M.R. (2013). Spatiotemporal linear mixed effects modeling for the mass-univariate analysis of longitudinal neuroimage data. NeuroImage.

[bb0025] Blencowe H., Cousens S., Oestergaard M.Z., Chou D., Moller A.-B., Narwal R., Adler A., Vera Garcia C., Rohde S., Say L., Lawn J.E. (2012). National, regional, and worldwide estimates of preterm birth rates in the year 2010 with time trends since 1990 for selected countries: a systematic analysis and implications. Lancet.

[bb0030] Boardman J.P., Counsell S.J., Rueckert D., Hajnal J.V., Bhatia K.K., Srinivasan L., Kapellou O., Aljabar P., Dyet L.E., Rutherford M.A., Allsop J.M., Edwards A.D. (2007). Early growth in brain volume is preserved in the majority of preterm infants. Ann. Neurol..

[bb0035] Boardman J.P., Craven C., Valappil S., Counsell S.J., Dyet L.E., Rueckert D., Aljabar P., Rutherford M.A., Chew A.T.M., Allsop J.M., Cowan F., Edwards A.D. (2010). A common neonatal image phenotype predicts adverse neurodevelopmental outcome in children born preterm. NeuroImage.

[bb0040] Cardoso M.J., Melbourne A., Kendall G.S., Modat M., Robertson N.J., Marlow N., Ourselin S. (2013). AdaPT: an adaptive preterm segmentation algorithm for neonatal brain MRI. NeuroImage.

[bb0045] Counsell S.J., Edwards A.D., Chew A.T.M., Anjari M., Dyet L.E., Srinivasan L., Boardman J.P., Allsop J.M., Hajnal J.V., Rutherford M.A., Cowan F.M. (2008). Specific relations between neurodevelopmental abilities and white matter microstructure in children born preterm. Brain.

[bb0050] Dubois J., Benders M., Borradori-Tolsa C., Cachia A., Lazeyras F., Ha-Vinh Leuchter R., Sizonenko S.V., Warfield S.K., Mangin J.F., Hüppi P.S. (2008). Primary cortical folding in the human newborn: an early marker of later functional development. Brain.

[bb0055] Dubois J., Benders M., Cachia A., Lazeyras F., Ha-Vinh Leuchter R., Sizonenko S.V., Borradori-Tolsa C., Mangin J.F., Hüppi P.S. (2008). Mapping the early cortical folding process in the preterm newborn brain. Cereb. Cortex.

[bb0060] Gilmore J.H., Lin W., Prastawa M.W., Looney C.B., Vetsa Y.S.K., Knickmeyer R.C., Evans D.D., Smith J.K., Hamer R.M., Lieberman J.A., Gerig G. (2007). Regional gray matter growth, sexual dimorphism, and cerebral asymmetry in the neonatal brain. J. Neurosci..

[bb0065] Gousias I.S., Edwards A.D., Rutherford M.A., Counsell S.J., Hajnal J.V., Rueckert D., Hammers A. (2012). Magnetic resonance imaging of the newborn brain: manual segmentation of labelled atlases in term-born and preterm infants. NeuroImage.

[bb0070] Han X., Pham D.L., Tosun D., Rettmann M.E., Xu C., Prince J.L. (2004). CRUISE: cortical reconstruction using implicit surface evolution. NeuroImage.

[bb0075] Herrmann L.R. (1976). Laplacian-isoparametric grid generation scheme. J. Eng. Mech. Div..

[bb0080] Hüppi P.S., Warfield S., Kikinis R., Barnes P.D., Zientara G.P., Jolesz F.A., Tsuji M.K., Volpe J.J. (1998). Quantitative magnetic resonance imaging of brain development in premature and mature newborns. Ann. Neurol..

[bb0085] Inder T.E., Warfield S.K., Wang H., Hüppi P.S., Volpe J.J. (2005). Abnormal cerebral structure is present at term in premature infants. Pediatrics.

[bb0090] Išgum I., Benders M.J.N.L., Avants B., Cardoso M.J., Counsell S.J., Gomez E.F., Gui L., Hüppi P.S., Kersbergen K.J., Makropoulos A., Melbourne A., Moeskops P., Mol C.P., Kuklisova-Murgasova M., Rueckert D., Schnabel J.A., Srhoj-Egekher V., Wu J., Wang S., de Vries L.S., Viergever M.A. (2015). Evaluation of automatic neonatal brain segmentation algorithms: the NeoBrainS12 challenge. Med. Image Anal..

[bb0095] Jones S.E., Buchbinder B.R., Aharon I. (2000). Three-dimensional mapping of cortical thickness using Laplace's equation. Hum. Brain Mapp..

[bb0100] Kapellou O., Counsell S.J., Kennea N., Dyet L., Saeed N., Stark J., Maalouf E., Duggan P., Ajayi-Obe M., Hajnal J., Allsop J.M., Boardman J., Rutherford M.A., Cowan F., Edwards A.D. (2006). Abnormal cortical development after premature birth shown by altered allometric scaling of brain growth. PLoS Med..

[bb0105] Kesler S.R., Vohr B., Schneider K.C., Katz K.H., Makuch R.W., Reiss A.L., Ment L.R. (2006). Increased temporal lobe gyrification in preterm children. Neuropsychologia.

[bb0110] Kuklisova-Murgasova M., Aljabar P., Srinivasan L., Counsell S.J., Doria V., Serag A., Gousias I.S., Boardman J.P., Rutherford M.A., Edwards A.D., Hajnal J.V., Rueckert D. (2011). A dynamic 4D probabilistic atlas of the developing brain. NeuroImage.

[bb0115] Lefevre J., Germanaud D., Dubois J., Rousseau F., de Macedo Santos I., Angleys H., Mangin J.-F., Huppi P.S., Girard N., De Guio F. (2015). Are developmental trajectories of cortical folding comparable between cross-sectional datasets of fetuses and preterm newborns?. Cereb. Cortex.

[bb0120] Lorensen W.E., Cline H.E. (1987). Marching Cubes: A High Resolution 3D Surface Construction Algorithm. Proceedings of the 14th Annual Conference on Computer Graphics and Interactive Techniques, SIGGRAPH'87.

[bb0125] Makropoulos A., Ledig C., Aljabar P., Serag A., Hajnal J.V., Edwards A.D., Counsell S.J., Rueckert D. (2012). Automatic tissue and structural segmentation of neonatal brain MRI using expectation–maximization. MICCAI Grand Challenge on Neonatal Brain Segmentation 2012 (NeoBrainS12).

[bb0130] Makropoulos A., Gousias I., Ledig C., Aljabar P., Serag A., Hajnal J., Edwards A., Counsell S., Rueckert D. (2014). Automatic whole brain MRI segmentation of the developing neonatal brain. IEEE Trans. Med. Imaging.

[bb0135] Mewes A.U.J., Hüppi P.S., Als H., Rybicki F.J., Inder T.E., McAnulty G.B., Mulkern R.V., Robertson R.L., Rivkin M.J., Warfield S.K. (2006). Regional brain development in serial magnetic resonance imaging of low-risk preterm infants. Pediatrics.

[bb0140] Moeskops P., Benders M.J.N.L., Pearlman P.C., Kersbergen K.J., Leemans A., Viergever M.A., Išgum I. (2013). Assessment of quantitative cortical biomarkers in the developing brain of preterm infants. SPIE Medical Imaging.

[bb0145] Moeskops P., Benders M.J.N.L., Kersbergen K.J., Groenendaal F., de Vries L.S., Viergever M.A., Išgum I. (2015). Development of cortical morphology evaluated with longitudinal MR brain images of preterm infants. PLoS One.

[bb0150] Murphy B.P., Inder T.E., Huppi P.S., Warfield S., Zientara G.P., Kikinis R., Jolesz F.A., Volpe J.J. (2001). Impaired cerebral cortical gray matter growth after treatment with dexamethasone for neonatal chronic lung disease. Pediatrics.

[bb0155] Nakagawa S., Schielzeth H. (2013). A general and simple method for obtaining r2 from generalized linear mixed-effects models. Methods Ecol. Evol..

[bb0160] Nishida M., Makris N., Kennedy D.N., Vangel M., Fischl B., Krishnamoorthy K.S., Caviness V.S., Grant P.E. (2006). Detailed semiautomated MRI based morphometry of the neonatal brain: preliminary results. NeuroImage.

[bb0165] Peterson B.S., Vohr B., Staib L.H., Cannistraci C.J., Dolberg A., Schneider K.C., Katz K.H., Westerveld M., Sparrow S., Anderson A.W., Duncan C.C., Makuch R.W., Gore J.C., Ment L.R. (2000). Regional brain volume abnormalities and long-term cognitive outcome in preterm infants. J. Am. Med. Assoc..

[bb0170] Peterson B.S., Anderson A.W., Ehrenkranz R., Staib L.H., Tageldin M., Colson E., Gore J.C., Duncan C.C., Makuch R., Ment L.R. (2003). Regional brain volumes and their later neurodevelopmental correlates in term and preterm infants. Pediatrics.

[bb0175] Pienaar R., Fischl B., Caviness V., Makris N., Grant P.E. (2008). A methodology for analyzing curvature in the developing brain from preterm to adult. Int. J. Imaging Syst. Technol..

[bb0180] Prastawa M., Gilmore J.H., Lin W., Gerig G. (2005). Automatic segmentation of MR images of the developing newborn brain. Med. Image Anal..

[bb0185] Rathbone R., Counsell S.J., Kapellou O., Dyet L., Kennea N., Hajnal J., Allsop J.M., Cowan F., Edwards A.D. (2011). Perinatal cortical growth and childhood neurocognitive abilities. Neurology.

[bb0190] Rodriguez-Carranza C.E., Mukherjee P., Vigneron D., Barkovich J., Studholme C. (2008). A framework for in vivo quantification of regional brain folding in premature neonates. NeuroImage.

[bb0195] Rueckert D., Sonoda L.I., Hayes C., Hill D.L., Leach M.O., Hawkes D.J. (1999). Nonrigid registration using free-form deformations: application to breast MR images. IEEE Trans. Med. Imaging.

[bb0200] Sadeghi N., Prastawa M., Fletcher P.T., Vachet C., Wang B., Gilmore J., Gerig G. (2013). Multivariate modeling of longitudinal MRI in early brain development with confidence measures. Proceedings/IEEE International Symposium on Biomedical Imaging: From nano to macro. IEEE International Symposium on Biomedical Imaging.

[bb0205] Serag A., Aljabar P., Ball G., Counsell S.J., Boardman J.P., Rutherford M.A., Edwards A.D., Hajnal J.V., Rueckert D. (2012). Construction of a consistent high-definition spatio-temporal atlas of the developing brain using adaptive kernel regression. NeuroImage.

[bb0210] Song Z., Awate S.P., Licht D.J., Gee J.C. (2007). Clinical neonatal brain MRI segmentation using adaptive nonparametric data models and intensity-based Markov priors. Med. Image Comput. Comput. Assist. Interv..

[bb0215] Srinivasan L., Dutta R., Counsell S.J., Allsop J.M., Boardman J.P., Rutherford M.A., Edwards A.D. (2007). Quantification of deep gray matter in preterm infants at term-equivalent age using manual volumetry of 3-tesla magnetic resonance images. Pediatrics.

[bb0220] Thompson D.K., Warfield S.K., Carlin J.B., Pavlovic M., Wang H.X., Bear M., Kean M.J., Doyle L.W., Egan G.F., Inder T.E. (2007). Perinatal risk factors altering regional brain structure in the preterm infant. Brain.

[bb0225] Thompson D.K., Wood S.J., Doyle L.W., Warfield S.K., Lodygensky G.A., Anderson P.J., Egan G.F., Inder T.E. (2008). Neonate hippocampal volumes: prematurity, perinatal predictors, and 2-year outcome. Ann. Neurol..

[bb0230] Van Leemput K., Maes F., Vandermeulen D., Suetens P. (1999). Automated model-based tissue classification of MR images of the brain. IEEE Trans. Med. Imaging.

[bb0235] Wang L., Shi F., Lin W., Gilmore J.H., Shen D. (2011). Automatic segmentation of neonatal images using convex optimization and coupled level sets. NeuroImage.

[bb0240] Wang L., Shi F., Yap P.-T., Gilmore J.H., Lin W., Shen D. (2012). 4D multi-modality tissue segmentation of serial infant images. PLoS One.

[bb0245] Wang L., Shi F., Li G., Gao Y., Lin W., Gilmore J.H., Shen D. (2013). Segmentation of neonatal brain MR images using patch-driven level sets. NeuroImage.

[bb0250] Wright R., Kyriakopoulou V., Ledig C., Rutherford M., Hajnal J., Rueckert D., Aljabar P. (2014). Automatic quantification of normal cortical folding patterns from fetal brain MRI. NeuroImage.

[bb0255] Xue H., Srinivasan L., Jiang S., Rutherford M., Edwards A.D., Rueckert D., Hajnal J.V. (2007). Automatic segmentation and reconstruction of the cortex from neonatal MRI. NeuroImage.

[bb0260] Yu X., Zhang Y., Lasky R.E., Datta S., Parikh N.A., Narayana P.A. (2010). Comprehensive brain MRI segmentation in high risk preterm newborns. PLoS One.

[bb0265] Zacharia A., Zimine S., Lovblad K.O., Warfield S., Thoeny H., Ozdoba C., Bossi E., Kreis R., Boesch C., Schroth G., Hüppi P.S. (2006). Early assessment of brain maturation by MR imaging segmentation in neonates and premature infants. Am. J. Neuroradiol..

